# Novel Carvacrol@activated Carbon Nanohybrid for Innovative Poly(lactide Acid)/Triethyl Citrate Based Sustainable Active Packaging Films

**DOI:** 10.3390/polym17050605

**Published:** 2025-02-24

**Authors:** Vassilios K. Karabagias, Aris E. Giannakas, Areti A. Leontiou, Andreas Karydis-Messinis, Dimitrios Moschovas, Nikolaos D. Andritsos, Apostolos Avgeropoulos, Nikolaos E. Zafeiropoulos, Charalampos Proestos, Constantinos E. Salmas

**Affiliations:** 1 Department of Food Science and Technology, University of Patras, 30100 Agrinio, Greece; vkarampagias@upatras.gr (V.K.K.); aleontiu@upatras.gr (A.A.L.); nandritsos@upatras.gr (N.D.A.); 2 Department of Material Science and Engineering, University of Ioannina, 45110 Ioannina, Greece; karydis.and@gmail.com (A.K.-M.); dmoschov@uoi.gr (D.M.); aavger@uoi.gr (A.A.); nzafirop@uoi.gr (N.E.Z.); 3 Laboratory of Food Chemistry, Department of Chemistry, National and Kapodistrian University of Athens Zografou, 15771 Athens, Greece; harpro@chem.uoa.gr

**Keywords:** poly(lactic acid), triethyl citrate, carvacrol, activated carbon, self-healable, active packaging, minced pork, shelf life

## Abstract

It has been well known for the past decade that the accumulation of food E-preservatives in the human body has harmful consequences for human health. Furthermore, scientists have realized that despite the convenience offered by petrochemical-derived polymers, a circular economy and sustainability are two current necessities; thus, the use of biodegradable alternative materials is imposed. The food packaging sector is one of the most rapidly changing sectors in the world. In recent years, many studies have focused on the development of active packaging films to replace old non-ecofriendly techniques with novel environmentally friendly methods. In this study, a novel self-healable, biodegradable active packaging film was developed using poly(lactic acid) (PLA) as a biopolymer, which was incorporated with a nanohybrid solid material as a natural preservative. This nanohybrid was derived via the absorption of carvacrol (CV) essential oil in an activated carbon (AC) nanocarrier. A material with a high carvacrol load of 71.3%wt. into AC via a vacuum-assisted adsorption method, functioning as a natural antioxidant and an antibacterial agent. The CV@AC nanohybrid was successfully dispersed in a PLA/triethyl citrate (TEC) matrix via melt extrusion, and a final PLA/TEC/xCV@AC nanocomposite film was developed. The study concluded that x = 10%wt. CV@AC was the optimum nanohybrid amount incorporated in the self-healable PLA/TEC and exhibited 277% higher ultimate strength and 72% higher water barrier compared to the pure PLA/TEC. Moreover, it remained ductile enough to show the slowest CV release rate, highest antioxidant activity, and significant antibacterial activity against *Staphylococcus aureus* and *Salmonella enterica* ssp. *enterica serovar Typhimurium*. This film extended the shelf life of fresh minced pork by four days, according to total viable count measurements, and decreased its lipid oxidation rate. Finally, this novel film preserved the nutritional value of porkby maintaining a higher heme iron content and showed a higher level of sensory characteristics compared to commercial packaging paper.

## 1. Introduction

Currently, the global transition from a linear to a circular green economy has imposed the use of alternative biodegradable materials, a global effort to reduce fossil fuels and their derivatives, and a global trend to replace chemical substances with other naturally derived materials exhibiting similar properties. These modern-age changes affect all industrial sectors [1,2,3,4,5]. The food packaging industrial sector has started using biodegradable natural biopolymers such as chitosan, cellulose, and starch, and polymers that can be produced from waste and byproducts, such as poly(lactic acid) (PLA) [3,6,7,8,9,10,11,12]. PLA is produced via the polymerization process of lactide acid (LA), which can be recovered from various food and agricultural byproducts, and thus, it is considered a cheap, environmentally friendly alternative precursor material for polymer matrix production [13,14,15]. Another advantage of PLA is its ability to be used in melting-blowing industrial processes to produce packaging films [16,17]. Thus, PLA could potentially be the replacement material for fossil-based polymers in the food packaging industry [10,12,18,19]. A disadvantage of PLA is its poor mechanical and thermomechanical properties [20]. To overcome this obstacle, scientists have treated PLA with various bio-based plasticizers [21,22,23,24,25,26,27]. Recently, triethyl-citrate (TEC) has been identified as a promising food-grade, bio-based plasticizer for PLA. This material improves PLA’s viscoelastic properties, and the PLA/TEC novel self-healable composite matrix [27] exhibited enhanced water/oxygen barrier properties and improved antioxidant/antibacterial activity.

Furthermore, the replacement of chemical additives with naturally derived bio-based antioxidant/antibacterial agents, such as natural extracts, essential oils (EOs), and their derivatives [28,29,30,31,32,33,34], has become a global trend. Carvacrol (CV) is the main component of oregano essential oil and is Generally Recognized as a Safe (GRAS) substance [35,36]. It has been widely used to develop active packaging films with various polymer or biopolymer matrixes because of its high antioxidant/antibacterial activity and its desirable odor for fresh meat products [37,38,39,40,41,42,43,44]. Another benefit of replacing such chemical additives is the avoidance of their harmful slow release into food [45,46,47]. Various technologies have been developed in the last decade for the encapsulation or nanoencapsulation of EOs and their derivatives in packaging films and the production of active packaging materials [48,49]. Such incorporation is promising and advantageous compared to the direct use of these substances for meat preservation [50,51]. The EO-based active packaging film method requires lower amounts of liquid and results in an extension of the shelf life of the meat and more acceptable aesthetic characteristics, such as color, odor, and texture. Moreover, it reduces consumer concerns regarding meat safety [37].

One of the most promising methods for incorporating EOs into polymeric matrixes for active packaging film development is their adsorption onto cheap, bio-based nanocarriers, such as nanoclays, natural zeolites, silicas, and activated carbons (AC). This adsorption method produces active nanohybrids that are sequentially dispersed in a polymer or biopolymer matrix [37,52,53,54,55,56,57,58,59,60,61,62]. AC has recently been used as a nanocarrier for EOs and their derivatives because of its low cost. This low cost is because it can be derived from various food and agricultural byproducts [63]. Its high specific surface area and micro/mesoporous structure make it an ideal nanocarrier for loading large amounts of liquid. This porous material also controls the release of adsorbed oil in food and maintains a low desorption rate [55,60,64,65,66]. Since AC exhibits pores of 1–100 nm, it can be called a nanomaterial [64,67]. The black color is considered favorable for packaging fresh meat products because it works as a light intrusion barrier and reduces food taint [68].

The development and characterization of a carvacrol@natural zeolite (CV@NZ) nanohybrid were presented in a recent study. A novel vacuum-assisted adsorption method was used to increase the oil content of this porous material. The maximum percentage of loading was up to 61 wt.%. The novel CV@NZ nanohybrid was incorporated into a self-healable PLA/TEC matrix to obtain a PLA/TEC/CV@NZ active film. Due to its high oil content, this film extended the shelf life of fresh minced pork to four days.

To continue this global effort to this research topic, this study investigates the development, advantages, and limitations of a novel food packaging film using almost completely naturally derived precursor materials. The first optimization of production process parameters was also carried out. A novel CV@AC nanohybrid was produced and extensively characterized using the above-mentioned adsorption method developed by our research team. This nanohybrid was incorporated into a PLA/TEC polymeric matrix via a melt extrusion process at 5, 10, and 15 wt. % nominal contents to obtain novel active film (PLA/TEC/xCV@AC, x = 5, 10, and 15). Pure AC was also incorporated with the same compositions in the same polymeric matrix to compare. The optimum novel active film was used to wrap the fresh pork minced meat and extend its shelf life. Such tests were carried out by monitoring the total variable counts, lipid oxidation, heme iron content, and sensory analysis during 10 days of storage at 4 °C. The innovative aspects of the current study are as follows: (1) the development and characterization of the CV@AC nanohybrid, (2) the development and characterization of both the novel PLA/TEC/xCV@AC and PLA/TEC/xAC films, and (3) the shelf-life experiment of fresh pork minced meat using the optimum PLA/TEC/xCV@AC as an active film. Future research steps targeting a final industrially produced material were also estimated.

## 2. Materials and Methods

### 2.1. Materials

PLA, with the tradename Ingeo™ Biopolymer 3052D, exhibited a crystalline melt temperature of 145–160 °C and glass transition temperature of 55–60 °C. It was purchased from NatureWorks LLC (Minnetonka, MN, USA). Liquid triethyl citrate (TEC) with an MW of 276.3 g/mol was purchased from Alfa Aesar GmbH & Co KG (Karlsruhe, Germany). Charcoal activated carbon (AC) with CAS Number 7440-44-0 (product number 31616-250g), soluble in water (0.5%) with a specific surface area (BET) of ≥800 m^2^/g, was purchased from Merck (Darmstadt, Germany). 2,2-Diphenyl-1-picrylhydrazyl (DPPH) was purchased from Sigma-Aldrich (Darmstadt, Germany). Absolute ethanol for analysis and acetate buffer (CH_3_COONa·3H_2_O) were purchased from Merck (Darmstadt, Germany).

### 2.2. Preparation of CV@AC Nanohybrid

The CV@AC nanohybrid was prepared using the vacuum-assisted method and the homemade apparatus, which was recently published [37].

### 2.3. Preparation of Films

For the preparation of all blends required for film production, a Mini Lab twin-screw extruder (Haake Mini Lab II, Thermo Scientific, ANTISEL, S.A., Athens, Greece) was used. The contents of PLA, TEC, pure AC, and CV@AC nanohybrid, as well as the twin extruder operating conditions (temperature and speed) and the sample code names, are listed in Table 1. All blends produced by the extrusion process were formed into films through a thermomechanical heat-pressing process using a hydraulic press with heated platens (Specac Atlas™ Series Heated Platens, Specac, Orpinghton, UK) at a constant pressure of 0.5 MPa and at 160 °C. An amount of 1.0 g from each blend was used to produce each film with an average diameter of approx. 11 cm and an average thickness in the range of 0.10–0.15 mm.

### 2.4. Physicochemical Characterization of CV@AC Nanohybrid and Pure AC Powders, as Well as of All Obtained Films

#### 2.4.1. CV Desorption Release Kinetics of CV@AC and PLA/TEC/xCV@AC Films

The estimation of the percentage of desorbed CV content and the release rate of this substance is crucial for CV@AC nanohybrids and PLA/TEC/xCV@AC films. Therefore, kinetic experiments were conducted using a moisture analyzer AXIS AS-60 (AXIS Sp. z o.o. ul. Kartuska 375b, 80-125 Gdańsk, Poland). The well-known pseudo-second-order adsorption-desorption equation was adopted for these interpretations [69,70]. The Arrhenius equations, theory [71,72,73], and methodology have been described in detail recently [74]. They are also presented in the Supplementary Material of the current study [37].

#### 2.4.2. X-Ray Diffraction (XRD) Analysis of CV@AC Nanohybrid and Pure AC Powders as Well as of All Obtained Films

To study the structural characteristics of the CV@AC nanohybrid and pure AC powders, as well as the PLA/TEC/xAC and PLA/TEC/xCV@AC films, XRD powder analysis measurements were performed in the range of 2 theta 0.5° to 40° using a Brüker XRD D8 Advance diffractometer (Brüker, Analytical Instruments, S.A., Athens, Greece).

#### 2.4.3. Fourier-Transform Infrared (FTIR) Analysis of CV@AC Nanohybrid and Pure AC Powders as Well as of All Obtained Films

To study the potential relaxation phenomena between the CV-adsorbed molecules, pure AC, CV@AC nanohybrid, and PLA/TEC matrix, FTIR spectroscopy measurements and analysis were carried out using an FT/IR-6000 JASCO Fourier-transform spectrometer (JASCO, Interlab, S.A., Athens, Greece).

#### 2.4.4. Scanning Electron Microscopy Images of Films

The morphological characteristics of all the prepared films were studied using a JEOL JSM-6510 LV SEM Microscope (JEOL Ltd., Tokyo, Japan).

#### 2.4.5. Instrumentation for Mechanical and Thermomechanical Properties of Films

All the developed films were measured according to the ASTM D638 method [75] using a Simantzü AX-G 5kNt instrument (Simantzü Asteriadis, S.A., Athens, Greece) to determine their tensile properties. The methodology was described in detail recently [27] and is also presented in the Supplementary Material.

### 2.5. Characterization of Active Packaging Properties of Films

#### 2.5.1. Instrumentation for Water/Oxygen Barrier Properties of Films

The water vapor transmission rate (WVTR) and the oxygen transmission rate (OTR) values of all PLA/TEC/xAC and PLA/TEC/xCV@AC films, as well as PLA//TEC film were determined according to the ASTM E96/E 96M-05 method [76] at 38 °C and 95% RH and ASTM D 3985 method [77] at 23 °C and 0% RH correspondingly. To achieve results independent of film thickness variation and easily comparable with other similar results, the water vapor diffusion coefficient (D_wv_) and oxygen permeability (Pe_O2_—cm^2^/s) values were calculated following Fick’s low theory and the methodology described in previous publications [74,78] and are presented in the Supplementary Material of this study.

#### 2.5.2. Antioxidant Activity of PLA/TEC/xCV@AC Films

Antioxidant activity measurements of each PLA/TEC/xCV@AC active film were carried out by determining the concentration required to obtain a 50% antioxidant effect (EC_50_) according to the methodology described in detail in a previously published study [37] and presented in the Supplementary Material of the current study.

#### 2.5.3. Instrumentation for Antibacterial Activity of PLA/TEC/xCV@AC Films

All obtained PLA/TEC/xCV@AC active films were tested for each antibacterial activity against one Gram-positive *Staphylococcus aureus* (NCTC 6571) and one Gram-negative *Salmonella enterica* subspecies *enterica* serovar Typhimurium (NCTC 12023) food pathogens. The experimental procedure followed for such measurements was based on the agar diffusion method, by measuring the diameters of the inhibition zones in the contact area of the films and around them with a Vernier caliper with 0.1 mm accuracy. The experimental procedure was repeated twice, and the films were measured in triplicate for each repetition.

### 2.6. Shelf Life of Fresh Pork Minced Meat

Packaging preservation test, lipid oxidation, heme iron, total variable counts measurements, as well as sensory analysis of Miced Pork meat wrapped with the optimum PLA/TEC/10CV@AC active film was performed according to the methodology described in previous papers [37] and also presented here in Supplementary Material.

### 2.7. Statistical Analysis

All data obtained from mechanical property measurements, water/oxygen barrier property measurements, EC_50_ values, TVC values, TBARS values, heme iron content values, and sensory analysis scores were subjected to statistical analysis to indicate any statistical differences. The non-parametric statistical Mood’s median test was chosen to evaluate the significance of the difference between the mean values of the properties. Assuming a significance level of *p* < 0.05, all measurements were conducted using three to five separate samples of each PLA/TEC/xAC and PLA/TEC/xCV@AC film. Statistical analyses were performed using SPSS software (v. 28.0, IBM, Armonk, NY, USA).

## 3. Results

### 3.1. Physicochemical Characterization of CV@AC Nanohybrid

**Figure 1:**
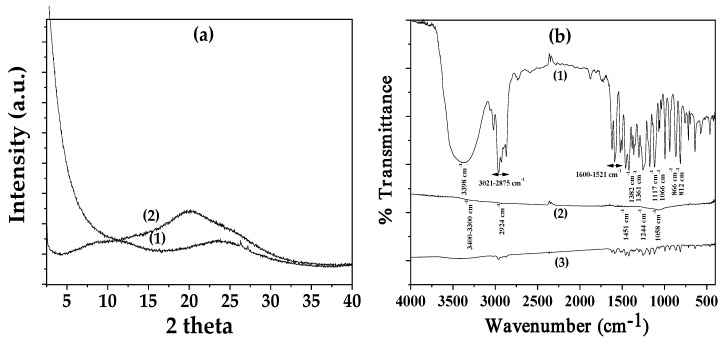
(**a**) XRD plots of (1) AC as received, and (2) CV@AC nanohybrid, and (**b**) FTIR plots of (1) pure CV, (2) AC as received, and (3) CV@AC nanohybrid.

Figure 1a presents the XRD plots of the AC as received (plot line (1)) and CV@AC nanohybrid (plot line (2)). In Figure 1b, the FTIR plots of these materials are presented with plot lines 2 and 3 correspondingly, while for pure CV, plot line (1) is relevant. The XRD plot of the AC as received powder exhibited a very broad peak at 2 theta around 24°, which is consistent with a typical amorphous material, while the amorphous broad peak of the CV@AC nanohybrid was shifted at 2 theta around 21°, indicating that the adsorption of CV subtly influenced the amorphous structure of pure AC.

**Figure 2:**
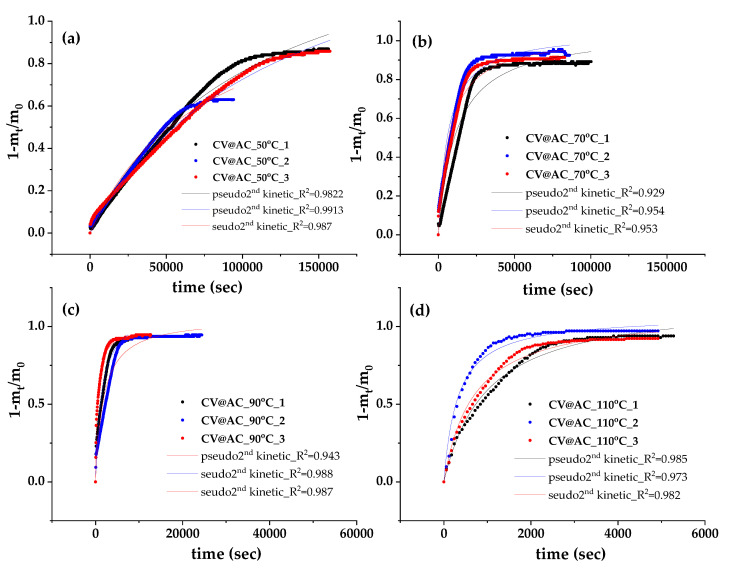
CV desorption isotherm kinetic plots (in triplicates) for CV@AC nanohybrid (**a**) 50 °C/323 K, (**b**) 70 °C/343 K, (**c**) 90 °C/363 K, and (**d**) 11 °C/383 K. The simulation plots according to the second-order pseudokinetic model are depicted by a red line diagram.

In the FTIR spectrum of pure CV (see plot line (1) in Figure 2b), the large broadband at 3398 cm^−1^ is assigned to the stretching vibration of the O–H functional group of the CV molecule [79,80,81]. The absorption bands at 2875–3021 cm^−1^ are assigned to the stretching vibrations of the aliphatic C–H groups of CV. The denoted absorption bands in the range of 1521–1600 cm^−1^ are assigned to C=C bond stretching vibrations of the aromatic ring of CV [79,80,81]. The bands at 1361 and 1382 cm^−1^ are assigned to the symmetric and asymmetric vibrations of CV’s isopropyl methyl group, respectively [79,80,81]. The bands at 812 and 866 cm^−1^ are assigned to the out-of-plane C–H wagging vibrations [79,80,81]. Finally, the bands in the range of 1066–1117 cm^−1^ are assigned to the ortho-substituted phenyl group [79,80,81].

The transmittance FTIR plot of AC as received (see plot line (2) in Figure 2b) has small peaks because of the high absorbance of AC’s black color. The band at 3400 cm^−1^ could be due to the –OH stretching or the presence of N-H groups [82,83]. The peak at 2924 indicates the presence of both methylene(–CH_2_–) bridges and aromatic C–H stretching vibrations [82,83]. The peak at 1451 cm^−1^ indicates the presence of C=C groups in the carbon. The additional small peaks at 1244 and 1058 cm^−1^ indicate the presence of S=O groups in the AC [82,83].

The FTIR plots of the CV@AC (see line (3) in Figure 1b) nanohybrid are a mixed plot of CV’s FTIR plots and AC’s FTIR plots. No shift in the CV reflection was observed, suggesting no or low-intensity interaction/relaxation between the AC and CV-adsorbed molecules [55].

The kinetics of the desorption process were extracted via plots of recorded values (1-m_t_/m_0_) versus time (t) for the CV@AC nanohybrid, as shown in Figure 2. The experiments were carried out at temperatures of 50 °C/323 K, 70 °C/343 K, 90 °C/363 K, and 11 °C/383 K. For each temperature, measurements were repeated three times for statistical reasons and are presented with different colored curves. To calculate the k_2_ and q_e_ mean values, these plots were fitted with the pseudo-second-order kinetic equation, which is mentioned in Equation (S2) in the Supplementary Materials. The results are presented in Table 2.

As shown in Table 2, as the temperature increases, both k_2_ and q_e_ values also increase, which means an increase in the CV release rate and in the total desorbed amount. The final percentage of the CV desorbed amount (%q_e_) was calculated to be 91.9% at 50 °C/323 K, 94.3% at 70 °C/343 K, 96.0% at 90 °C/363 K, and 98.9% at 110 °C/383 K. Thus, AC seems to be a very promising nanocarrier for EO-based bioactive compounds as it manages to carry and release high amounts of CV. This is probably due to its high specific surface area (≥800 m^2^/g).

**Figure 3:**
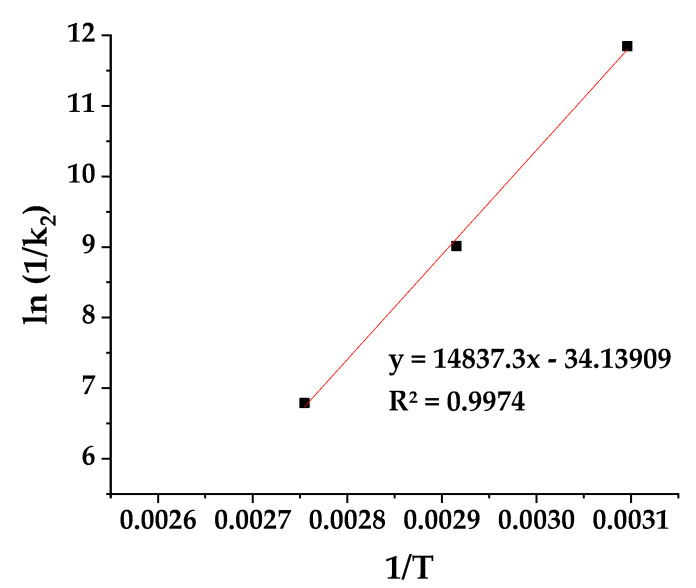
Plot of ln(1/k_2_) values as a function of (1/T) for the CV@AC nanohybrid.

The ln(1/k_2_) values were determined using the calculated k_2_ values and are plotted as a function of (1/T) for the CV@AC nanohybrid in Figure 3.

The estimated slope of the linear fitted plot in Figure 3 was fed to Equations (S4) and (S5) in the Supplementary Materials to determine the CV desorption energy (E_0,des_) from the CV@AC nanohybrid. Thus, the calculated E_0des_ is 29.5 Kcal/mol clearly indicating the chemisorption of CV in the AC nanocarrier according to the Arrhenius theory.

### 3.2. Physicochemical Characterization of Films

**Figure 4:**
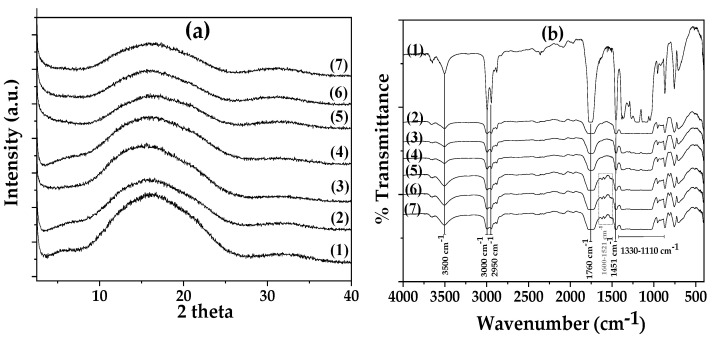
(**a**) XRD plots and (**b**) FTIR plots of (1) pure PLA/TEC, (2) PLA/TEC/5AC, (3) PLA/TEC/10AC, (4) PLA/TEC/15AC, (5) PLA/TEC/5CV@AC, (6) PLA/TEC/10CV@AC, and (7) PLA/TEC/15CV@AC films.

Both the XRD (Figure 4a) and FTIR plots (Figure 4b) of all developed films are presented in Figure 4.

As it is observed in Figure 4a, the XRD plot of the pure PLA/TEC film (see plot line (1)) has a broad peak at 2-theta of around 15.5°, which corresponds to its amorphous crystal phase [27]. Furthermore, the addition of pure AC to the PLA/TEC matrix did not affect the crystallinity of any of the obtained PLA/TEC/xAC films (see plot lines (2), (3), and (4) in Figure 4a). In addition, all the obtained plots of the PLA/TEC/xCV@AC films (see plot lines (5), (6), and (7) in Figure 4a) are similar to that of the pure PLA/TEC film and correspond to an amorphous polymer matrix.

In Figure 4b, the FTIR plots of pure PLA/TEC film and all obtained PLA/TEC/xAC and PLA/TEC/xCV@AC films are presented. As shown recently, TEC is an excellent plasticizer for PLA because of its chemical similarity to PLA functional groups [26,27,84]. Thus, the FTIR plot of the pure PLA/TEC film (see line (1) in Figure 4b) is a mixture of PLA and TEC bands of similar functional groups. The bands at 3500 cm^−1^ and 3496 cm^−1^ are assigned to the hydroxyl (O–H) group of PLA and TEC, correspondingly [26,27]. The bands at 3000, 2950, and 3050 cm^−1^ and those at 2985, 2941, and 2909 cm^−1^ are assigned to the asymmetrical and symmetrical stretching of the methyl group (–C–H) of PLA and TEC, correspondingly [26,27]. The bands at 1760 cm^−1^ and 1741 cm^−1^ are assigned to the stretching vibration of the carbonyl group (C=O) from the ester units of PLA and TEC, correspondingly [26,27]. The bands at 1110–1330 cm^−1^ are assigned to the stretching vibrations of the ether (–C–O–) groups of PLA and TEC. Finally, the band at 1451 cm^−1^ is assigned to the bending vibrations of the methyl (–CH_3_) group of PLA.

In the FTIR plots of all PLA/TEC/xAC films (see plot lines (2), (3), and (4) in Figure 4a), and all PLA/TEC/xAC@CV (see plot lines (5), (6), and (7) in Figure 4a), the addition of both black colored AC and CV@AC powders decreases the intensity of transmittance. The addition of pure AC does not add any additional peaks in the obtained FTIR plots of all PLA/TEC/xAC films (see plot lines (2), (3), and (4) in Figure 4a) and does not cause any shifting to the PLA/TEC existed peaks suggesting the compatibility of pure AC with PLA/TEC matrix [84].

In the FTIR plots of all PLA/TEC/xAC@CV (see plot lines (5), (6), and (7) in Figure 4a) films, a group of small peaks in the range of 1600–1521 cm^−1^ indicates the presence of encapsulated CV molecules.

**Figure 5:**
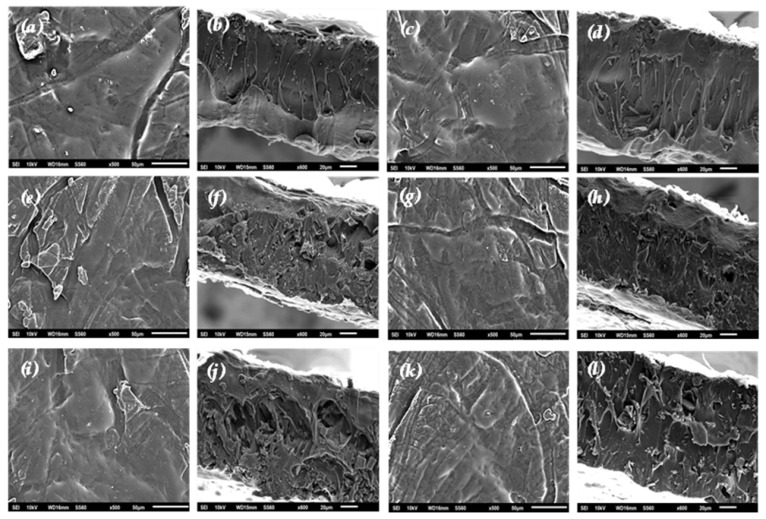
SEM images of surface (**a**,**c**,**e**,**g**,**i**,**k**) and cross-section (**b**,**d**,**f**,**h**,**j**,**l**) nanocomposite films of PLA/TEC/5AC (**a**,**b**), PLA/TEC/5CV@AC (**c**,**d**), PLA/TEC/10AC (**e**,**f**), PLA/TEC/10CV@AC (**g**,**h**), PLA/TEC/15AC (**i**,**j**), PLA/TEC/15CV@AC (**k**,**l**).

In Figure 5, the SEM images of the surface and cross-section morphologies of the films are presented.

Both pure AC and the CV@AC nanohybrid were homogeneously dispersed in the PLA/TEC matrix, indicating their enhanced compatibility, which improved the behavior of the obtained films.

The surface and relative cross-sectional images of the nanocomposite films of PLA/TEC/AC and PLA/TEC/CV@AC with different ratios (5, 10, and 15% wt) of CV@AC nanohybrid and pure AC (white dots can be clearly identified in the PLA/TEC matrix) are shown in Figure 5. All the obtained films were uniform in thickness. By increasing the contents of both AC and CV@AC, the degree of dispersion increases. The SEM micrographs with lower amounts of AC (5%wt) showed surface and cross-section morphologies with low dispersion and a continuous phase without heterogeneities. Some aggregates and voids were observed in the cross-sectional film, where pure AC with a higher concentration (15% wt.) had accumulated.

It should be mentioned that based on the SEM (surface and cross-sectional) studies of all samples, a significant difference was observed when the CV@AC hybrid nanostructure was incorporated into the polymer matrix of PLA/TEC, as better interfacial adhesion was evident compared to the corresponding pure AC nanocomposite film.

Overall, it is concluded that the optimal nanocomposite active packaging film is achieved at 10 wt% CV@AC concentration in the polymer matrix, as the nanohybrid can be better stabilized without agglomeration with complete mixing and homogeneous morphology.

### 3.3. Results for Mechanical and Thermomechanical Properties of Films

From the obtained stress-strain curves, the Elastic Modulus E (MPa), ultimate strength σ_uts_ (MPa), and % elongation at break values were calculated for all tested films and are listed in Table 3 for comparison.

**Figure 6:**
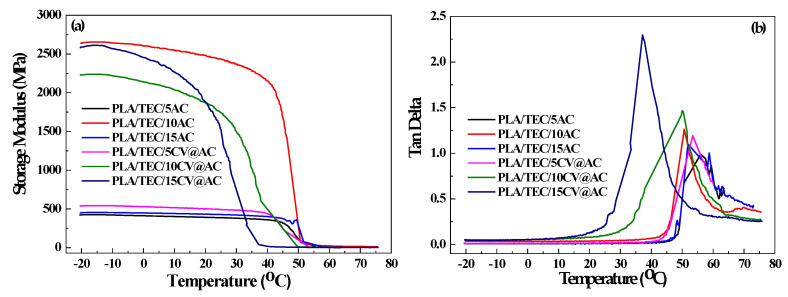
(**a**) Storage modulus plots, (**b**) Tan delta plots of all PLA/TEC/xAC and PLA/TEC/xCV@AC packaging films, as well as the pure PLA/TEC film.

The data from the dynamic mechanical analysis (DMA) measurements, storage modulus, and tan delta for all tested PLA/TEC/xAC and PLA/TEC/xCV@AC films, as well as the pure PLA/TEC film, are plotted in Figure 6a,b.

As it is depicted in Figure 6, medium (10%wt.) and high (15%wt.) CV@AC loading contents increase the storage modulus of obtained PLA/TEC/10CV@AC and PLA/TEC/15CV@AC composite films while low (5%wt.) CV@AC loading does not significantly affect the storage modulus of the obtained PLA/TEC/5AC@AC.

### 3.4. Water/Oxygen Barrier Properties of Films

In Table 4 are listed the water vapor transmission rate (WVTR), oxygen transmission rate (OTR), water vapor diffusion coefficient (D_wv_), and oxygen permeability Pe_O2_ mean values of all tested PLA/TEC/xAC and PLA/TEC/xCV@AC films, as well as the pure PLA/TEC film for comparison.

### 3.5. Antioxidant Activity of PLA/TEC/xCV@AC Films

Table 5 shows the calculated EC_50_ mean values for all obtained PLA/TEC/xCV@AC active films.

As shown in Table 5, the lowest EC_50_ value was observed for the film containing 10%wt. CV@AC nanohybrid.

### 3.6. CV Release Kinetics of PLA/TEC/xCV@AC Films

**Figure 7:**
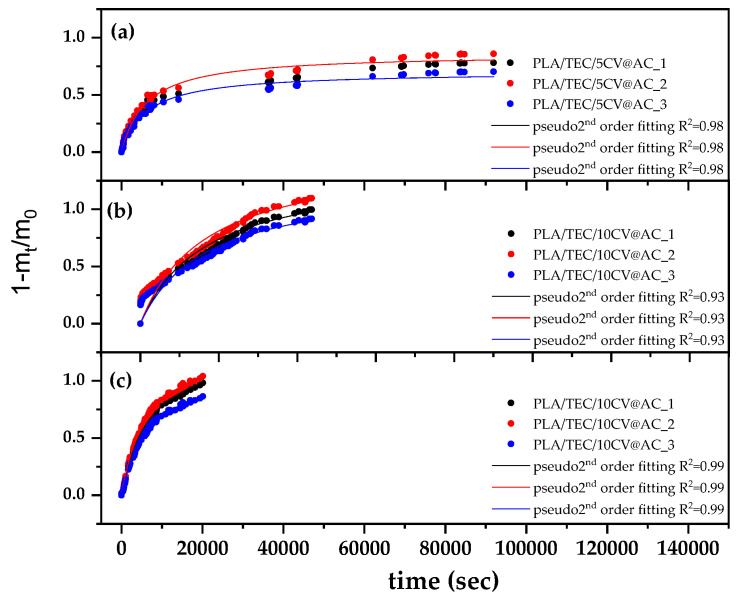
CV desorption isotherm kinetic plots (in triplicates) for (**a**) PLA/TEC/5CV@AC, (**b**) PLA/TEC/10CV@AC, and (**c**) PLA/TEC/15CV@AC films.

The (1-m_t_/m_0_) values as a function of time (t) for all PLA/TEC/xCV@AC active films (in triplicate) at 70 °C are shown in Figure 7.

The data were fitted with a pseudo-second-order kinetic model (see red line plots in Figure 7) to calculate the k_2_ and q_e_ values using Equation (S1). The resulting k_2_ and q_e_ mean values are listed in Table 6 for all PLA/TEC/xCV@AC active films.

As shown in Table 6, as the CV@AC content increases, the calculated k_2_ values decrease, while the calculated q_e_ values increase. This means that by increasing the CV@AC nanohybrid loading in the PLA/TEC matrix, the CV release rate decreases, and the CV-loaded content increases. All PLA/TEC/xCV@AC active films desorbed a significant amount of adsorbed CV. Both PLA/TEC/10CV@AC and PLA/TEC/15CV@AC active films desorbed almost all of the adsorbed CV. The results reported here for k_2_ and q_e_ values rates are in agreement with similar reports, implying that by increasing the loaded EOs derivative amount in a polymer matrix diffusivity of such EOs derivatives molecules inside polymer film decreases [37,53,55,85]. Comparing the k_2_ and q_e_ calculated values for PLA/TEC/xCV@AC active films with the recently reported values for PLA/TEC/xCV@NZ active films, it is observed that PLA/TEC/xCV@AC films desorbed higher amounts of CV at lower release rates than PLA/TEC/xCV@NZ films [37]. From the estimated k_2_ and q_e_ values, it is concluded that PLA/TEC/10CV@AC active film releases all of its adsorbed CV (up to 99%) at the lowest release rate. High-release amounts with a slow-release rate are beneficial for such active packaging films.

### 3.7. Antibacterial Activity of PLA/TEC/xCV@AC Films

The results of the antibacterial test of all PLA/TEC/xCV@AC active films against *S. aureus* and *S. typhimurium* are listed in Table 7.

As shown in Table 7, all PLA/TEC/xCV@AC films exhibited significant antibacterial activity against both *Staphylococcus aureus* and *Salmonella enterica* spp. *enterica serovar typhimurium*, pathogenic bacteria in the contact area. As recently shown, pure PLA/TEC films exhibited strong antibacterial activity against Gram-negative pathogenic bacteria in all six replicates, but it was not as effective against Gram-positive pathogenic bacteria, showing moderate antibacterial activity in three out of six replicates [27]. The results here suggest that the incorporation of CV@AC nanohybrid PLA/TEC-based films enhances the antibacterial activity.

### 3.8. Preservation of Fresh Minced Pork Meat

#### 3.8.1. Microbiological Evaluation of Minced Pork

**Figure 8:**
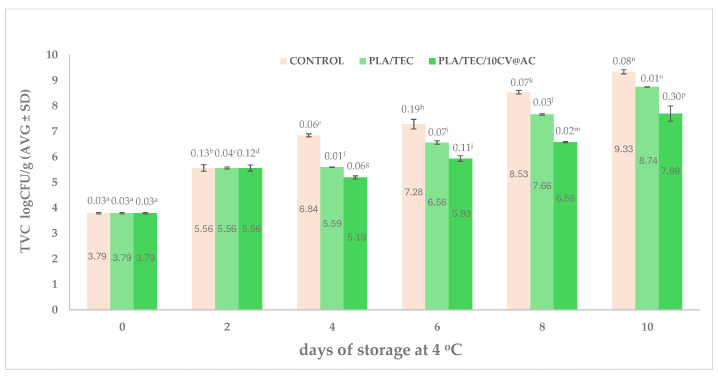
Column bar diagram of calculated TVC mean values for minced pork wrapped with the commercial Ayfantis company package (Control), PLA/TEC, and PLA/TEC/10CV@AC films during the 10 days of storage at 4 ± 1 °C. Different letters in each column indicate statistically significant differences at a confidence level of *p* < 0.05 (see also Figure S7 and Table S4).

Figure 8 shows the calculated TVC values for minced pork wrapped with the commercial Aifantis company package (Control) (Aifantis Group—HeadQuarters, Acheloos Bridge, Agrinio, Greece) PLA/TEC, and PLA/TEC/10CV@AC films.

A higher increase in TVC values (see Figure 8) was observed for minced pork wrapped with commercial film (control sample). This sample reached the limit of acceptance for fresh pork meat, according to the International Commission on Microbiological Specifications for Foods (ICMFS), 7 log CFU/g, after the 4th day of storage. Minced pork wrapped with PLA/TEC film reaches the 7 log CFU/g after the 6th day of storage. This result is in accordance with a recent report, which showed that PLA/TEC has antioxidant and antibacterial activities due to the presence of TEC [27]. PLA/TEC/10CV@AC active film reached a 7 log CFU/g value after the 8th day of storage, indicating the lowest TVC increase rate. This indicates a shelf-life extension of four days from a microbiological point of view compared to the control sample. The shelf-life extension of minced pork is equal to that reported recently using PLA/TEC/xCV@NZ active films [37].

#### 3.8.2. Lipid Oxidation and Heme Iron Contents of Minced Pork

**Figure 9:**
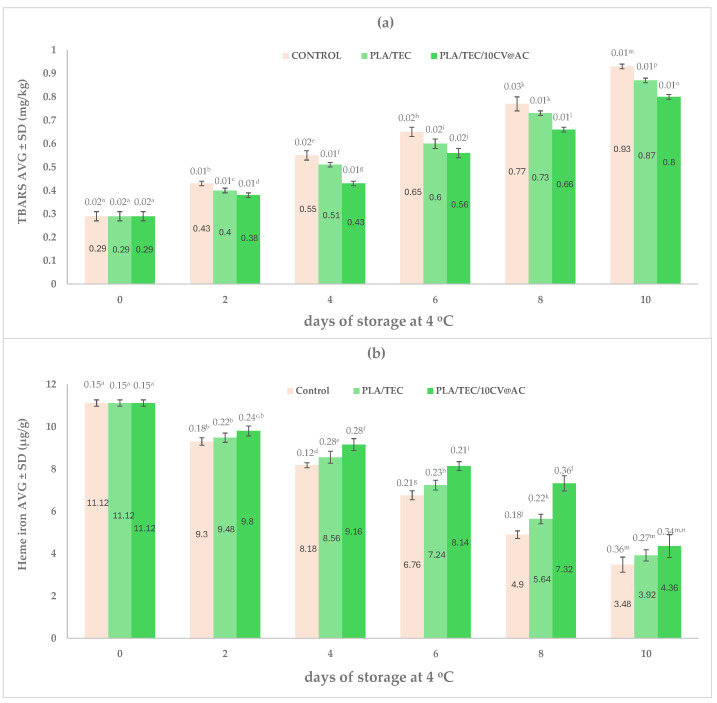
(**a**) TBARS mean values and (**b**) heme iron mean values for minced pork wrapped with the commercial Ayfantis package (Control), PLA/TEC, and PLA/TEC/10CV@AC films during 10 days of storage at 4 ± 1 °C. Different letters in each column indicate statistically significant differences at a confidence level of *p* < 0.05 (see also Figures S8 and S9 and Tables S5 and S6).

Figure 9 shows the calculated mean values of TBARS along with the calculated heme iron mean values of minced pork wrapped with the commercial Ayfantis company package (Control), PLA/TEC, and PLA/TEC/10CV@AC films.

The results show that both PLA/TEC and PLA/TEC/10CV@AC active films exhibited lower TBARS increment rates than the commercial film (control sample). PLA/TEC/10CV@AC active film had the lowest TBARS increment rate. Thus, on the 8th day, when minced pork wrapped in the PLA/TEC/10CV@AC film almost exceeded the limit of acceptance for TVC, 7 logCFU/g, minced pork had a TBARS value that was 14% lower than that of minced pork wrapped with the commercial film.

At the same time, both PLA/TEC and PLA/TEC/10CV@AC active films exhibited lower heme iron decrement rates than the commercial film (see Figure 9b). The lowest heme iron decrement rate was recorded for the PLA/TEC/10CV@AC active film. This result is consistent with the lowest TBARS value for the PLA/TEC/10CV@AC active film. Thus, PLA/TEC/10CV@AC prevents minced pork from lipid oxidation deterioration in minced pork while maintaining the highest heme iron content. In addition, a linear correlation between the increasing TBARS and the decreasing heme iron content values was recorded in accordance with all previous reports [54,55,85].

#### 3.8.3. Sensory Analysis of Minced Pork

The sensory analysis results of the odor, color, and texture of minced pork wrapped in PLA/TEC and PLA/TEC/10CV@AC films, as well as in a commercial film (control sample), are listed in Table 8 for comparison.

As observed in Table 8, both pure PLA/TEC and PLA/TEC/10CV@AC active films achieved much better and acceptable odor, color, and texture values than commercial packaging films. PLA/TEC/10CV@AC film exhibited the highest sensory analysis values during the 10 days of storage.

## 4. Discussion

In this work, we developed and characterized a novel CV@AC nanostructure, which was subsequently utilized as a nano-reinforcement and CV control release nanocarrier to develop novel PLA/TEC/xCV@AC (x = 5, 10, 15%wt.) active packaging films. PLA/TEC/xAC (x = 5, 10, 15%wt.) films were also developed for comparison. To the best of our knowledge, CV@AC nanostructures and PLA/TEC/xAC and PLA/TEC/xCV@AC packaging films were developed, characterized, and reported for the first time. Using the vacuum-assisted adsorption method, a high amount of CV was loaded in AC, and the CV desorption kinetics experiments revealed that this CV@AC nanostructure achieved the release of high amounts of CV up to 98.9 wt.%. This CV release desorbed amount was higher than 61.7 wt.% of the CV@NZ nanostructure reported by a previous study [37]. Desorption kinetic experiments also suggested the chemi-physisorption of CV on the AC nanocarrier, in accordance with the XRD and FTIR characterization of the CV@AC nanostructure.

For PLA/TEC/xAC and PLA/TEC/xCV@AC (x = 5, 10, 15%wt.) active packaging films were prepared, and SEM studies confirmed that both pure AC and the CV@AC nanostructure were homogeneously dispersed, indicating their enhanced compatibility with the PLA/TEC matrix. Tensile property studies show that pure AC enhances the ultimate strength and decreases the ductility of the obtained PLA/TEC/xAC films, while the CV@AC nanostructure enhances the ultimate strength and preserves the ductility of the obtained PLA/TEC/xCV@AC films. This indicates that pure AC serves as a reinforcement of the PLA/TEC matrix, while the CV@AC nanostructure serves as both a reinforcement and plasticizer in the PLA/TEC matrix. The increase in mechanical strength by increasing the AC-loaded content on the PLA/TEC matrix is in accordance with previous reports where activated carbon was added to chitosan, starch, and cellulose-based packaging films [86,87,88]. The addition of both pure AC and CV@AC nanostructures in the PLA/TEC matrix increased the water barrier properties, in accordance with previous reports where AC was added to starch- and chitosan-based films [86,89]. On the other hand, the oxygen barrier increases for lower (5%wt.) both AC and CV@AC loaded contents and remains constant for medium (10%wt.) AC and CV@AC loaded contents and decreases for higher (15%wt.) AC and CV@AC loaded contents. No data are available in the literature on the oxygen barrier properties of AC-based films for comparison. CV release desorption kinetics of PLA/TEC/xCV@AC films reveal that an increment of CV@AC loaded contents decreases CV release rates in accordance with SEM studies, which show that an increase of CV@AC loaded contents increases particle aggregation. This CV@AC aggregation decreases CV diffusivity inside the PLA/TEC matrix, thus, the highest antioxidant activity (lowest EC_50_ value) was recorded for the PLA/TEC/10CV@AC active film.

Overall, considering that the aim of this study was to add the CV@AC nanohybrid to the PLA/TEC matrix to obtain the highest possible CV content (%wt.) and, at the same time, to improve as many packaging properties as possible, we conclude that PLA/TEC/10CV@AC active film is optimal. Therefore, the PLA/TEC/10CV@AC film has: (i) 277% higher ultimate strength than PLA/TEC and remains ductile, (ii) 72% higher water barrier than the PLA/TEC film, (iii) almost the same oxygen barrier properties as the PLA/TEC film, (iv) low CV release rate (k_2_ value), (v) the highest antioxidant activity, (vi) significant antibacterial activity against Gram+ and Gram- pathogens, and is self-healable (see Video S1 in Supplementary Materials). The shelf life of minced pork wrapped with this film was extended by four days compared to that of minced pork wrapped in commercial paper, as shown by the recorded TVC values. Minced pork wrapped with the PLA/TEC/10CV@AC active film showed the lowest lipid oxidation rates (TBARS values) and maintained the highest heme iron content and sensory characteristics after 10 days of storage.

This work, along with a recently reported study, is a state-of-the-art study on how EO derivatives such as CV can be encapsulated in cheap, naturally abundant, and edible nanocarriers such as NZ and AC and dispersed in biodegradable self-healable active packaging films with controlled release antioxidant/antibacterial properties for fresh meat shelf life extension [85]. This technology is promising and advantageous over the direct use of EOs and their derivatives for meat preservation, as it requires lower amounts of EOs and results in an extension of the shelf life of the meat with more acceptable aesthetic characteristics, such as color, odor, and texture [50,51,90,91,92]. This technology is also advantageous over the direct incorporation of CV into PLA films, as it results in slower release rates and longer shelf life of meat [93]. The antioxidant activity mechanism of EOs, such as CV, through the regeneration of reactive oxygen species, is well established [94]. EOs such as CV antibacterial mechanism take place through the easy penetration of CV’s molecule in the lipids of the cell membrane, the disruption of the cell membrane, which causes loss of membrane integrity and cellular contents, and finally leads to cell death [95]. Through the adsorption of CV molecules on AC nanostructures and the incorporation of CV@AC nanohybrid in the PLA/TEC matrix, CV molecules were released slowly and in small amounts in the minced meat, rregenerating reactive oxygen species, which prevented lipid oxidation in minced pork and reduced heme iron content. Simultaneously, these slow-release CV molecules penetrate food pathogen cells and prevent minced pork from microbiological deterioration for a longer period of time with less alteration of its sensory characteristics.

## 5. Conclusions

Overall, this study indicates a novel alternative, biodegradable, self-healable active packaging film with an optimum dispersed amount of 10% wt. CV@AC nanohybrid in a PLA/TEC matrix. This material was produced via an extrusion molding method, which is commonly used in industrial processes. This film with the code name PLA/TEC/10CV@AC exhibited improved antioxidant/antibacterial activity and slow CV release rates and succeeded in extending the shelf life of fresh minced pork, according to TVC values, for four days. Furthermore, the determination of TBARS and heme iron content showed that this film delayed lipid oxidation and correspondingly provided higher nutritional values of fresh minced pork. This meat also exhibited acceptable or improved sensory characteristics than the meat packaged using commercial paper. Thus, this film shows great potential for use as an innovative, sustainable, biodegradable, self-healable active film with the capability to extend the shelf life of fresh meat products.

Although this study seems to have obtained a novel advantageous food packaging film, there are limitations in the use of such precursor materials for film preparation. For example, AC is approved by many organizations as a process aid material but not as a food ingredient that is directly added. Thus, further measurements of AC migration into food are needed. Moreover, not all ACs are approved for use in food preservation because of chemicals, heavy metals, or acid residues that can pose health risks. AC content is a critical parameter because, at the same time, it can absorb both harmful and beneficial compounds. It may remove essential nutrients, flavors, and natural antioxidants, thereby reducing food quality, color, and taste. Similar limitations are observed in the use of CV. Additionally, the use of this substance should follow the directions of regulatory agencies (e.g., FDA, EFSA), which limit the allowable concentration of carvacrol in foods due to its potential toxicity at high doses. High concentrations can cause irritation of the digestive system and may not be safe for long-term consumption. The cost, processing challenges, and availability of food-derived extracts make pure carvacrol or carvacrol-rich essential oils expensive compared to traditional preservatives.

All the above-mentioned limitations could be addressed in future work before scale-up calculations for final industrial production.

## Figures and Tables

**Table 1:** Contents of PLA, TEC, pure AC, and CV@AC nanohybrid, as well as the twin extruder operating conditions (temperature and speed) used for the preparation of all obtained blends.

Sample Name	PLA (g)	TEC (mL-% *v*/*w*)	AC (g-% *w*/*w*)	CV@AC (g-% *w*/*w*)	Twin Extruder Operating Conditions		
					T (°C)	Speed (rpm)	Time (min)
PLA/TEC	4	0.6–15	-	-	180	120	5
PLA/TEC/5AC	4	0.6–15	0.2–5	-	180	120	5
PLA/TEC/10AC	4	0.6–15	0.4–10	-	180	120	5
PLA/TEC/15AC	4	0.6–15	0.6–15	-	180	120	5
PLA/TEC/5CV@AC	4	0.6–15	-	0.2–5	180	120	5
PLA/TEC/10CV@AC	4	0.6–15	-	0.4–10	180	120	5
PLA/TEC/15CV@AC	4	0.6–15	-	0.6–15	180	120	5

**Table 2:** Calculated k_2_ and q_e_ mean values from CV desorption kinetic plots for CV@AC nanohybrid.

CV@AC	50 °C/323 K	70 °C/343 K	90 °C/363 K	110 °C/383 K
k_2_ (s^−1^)	7.17 × 10^−6^ ± 1.97 × 10^−6^	1.22 × 10^−4^ ± 3.53 × 10^−5^	1.12 × 10^−3^ ± 9.67 × 10^−4^	1.73 × 10^−3^ ± 1.2 × 10^−4^
q_e_	0.919 ± 0.006	0.943 ± 0.010	0.960 ± 0.006	0.989 ± 0.006

**Table 3:** Elastic Modulus E (MPa), ultimate strength σ_uts_ (MPa), and % elongation at break values were calculated for all PLA/TEC/xAC and PLA/TEC/xCV@AC active films, as well as for the pure PLA/TEC film.

Sample Name	E (Mpa)	σ_uts_ (MPa)	%ε
PLA/TEC	564.1 ± 93.7 ^a^	8.0 ± 1.9 ^a^	436.3 ± 285.6 ^a^
PLA/TEC/5 AC	3391.9 ± 228.7 ^b^	59.7 ± 7.4 ^b^	3.1 ± 0.5 ^b^
PLA/TEC/10 AC	2498.7 ± 538.1 ^c^	42.6 ± 4.9 ^c^	2.6 ± 0.6 ^b^
PLA/TEC/15 AC	2881.7 ± 683.5 ^d,c^	50.7 ± 7.6 ^d,b^	2.9 ± 0.5 ^b^
PLA/TEC/5CV@AC	2003.5 ± 279.3 ^d,c^	34.6 ± 9.7 ^d,b,c^	15.9 ± 24.7 ^b^
PLA/TEC/10CV@AC	1736.7 ± 467.7 ^d,c^	30.2 ± 4.7 ^e,d,b,c^	171.3 ± 58.0 ^a,c^
PLA/TEC/15CV@AC	625.0 ± 267.0 ^a^	8.8 ± 1.9 ^a^	367.7 ± 34.4 ^d,a^

Different letters in each column indicate statistically significant differences at a confidence level of *p* < 0.05 (see also Figure S1 and Table S1).

**Table 4:** Water vapor transmission rate (WVTR) and oxygen transmission rate (OTR) mean values, as well as the calculated water vapor diffusion coefficient (D_wv_) and oxygen permeability Pe_O2_ mean values of all tested films.

	Thickness (mm)	WVTR (g·cm^−2^·s^−1^) × 10^−6^	D_wv_ (cm^2^·s^−1^) × 10^−4^	Thickness (mm)	OTR (mL·m^−2^·day^−1^)	P_eO2_ (cm^2^·s^−1^) × 10^−9^
PLA/TEC	0.14 ± 0.01	1.8 ± 0.67	5.71 ± 1.69 ^a^	0.08 ± 0.01	284.6 ± 56.2	2.63 ± 0.55 ^a^
PLA/TEC/5 AC	0.11 ± 0.02	0.83 ± 0.21	1.57 ± 0.75 ^b^	0.12 ± 0.04	151.5 ± 27.6	2.12 ± 0.31 ^a^
PLA/TEC/10 AC	0.13 ± 0.01	0.37 ± 0.05	1.13 ± 0.05 ^b^	0.15 ± 0.01	121.0 ± 14.1	2.04 ± 0.03 ^b,a^
PLA/TEC/15 AC	0.12 ± 0.02	0.58 ± 0.19	1.56 ± 0.17 ^c,b^	0.13 ± 0.01	159.3 ± 55.1	2.39 ± 1.20 ^a^
PLA/TEC/5CV@AC	0.10 ± 0.02	0.54 ± 0.28	1.14 ± 0.54 ^b^	0.14 ± 0.02	137.0 ± 39.6	2.23 ± 0.35 ^a^
PLA/TEC/10CV@AC	0.10 ± 0.03	0.94 ± 0.28	1.60 ± 0.92 ^b^	0.09 ± 0.01	268.0 ± 152.7	2.79 ± 1.14 ^a^
PLA/TEC/15CV@AC	0.13 ± 0.01	0.65 ± 0.22	1.46 ± 0.52 ^b^	0.08 ± 0.01	361.5 ± 87.0	3.43 ± 1.37 ^a^

Different letters in each column indicate statistically significant differences at the confidence level of *p* < 0.05 (see also Figure S2 and Table S2).

**Table 5:** Calculated EC_50_ mean values for all PLA/TEC/xCV@AC active films.

Sample	EC_50_ (mg/L)
PLA/TEC/5CV@AC	14.39 ± 0.60
PLA/TEC/10CV@AC	6.72 ± 1.23
PLA/TEC/15CV@AC	10.47 ± 0.43

**Table 6:** Calculated k_2_ and q_e_ mean values for all PLA/TEC/xCV@AC active films.

	k_2_ (s^−1^)	q_e_
PLA/TEC/5CV@AC	0.000238 ± 0.000055	0.78 ± 0.08
PLA/TEC/10CV@AC	0.0000441 ± 0.0000032	0.99 ± 0.01
PLA/TEC/15CV@AC	0.000117 ± 0.000011	0.97 ± 0.01

**Table 7:** Antibacterial activity results of all PLA/TEC/xCV@AC films against *S. aureus* and *S. typhimurium*.

Sample	*S. aureus*	*S. typhimurium*
	No. of 6 replicates with growth in the contact area of the sample	No. of 6 replicates with growth in the contact area of the sample
PLA/TEC/5CV@AC	0/6 ^a^	0/6 ^a^
PLA/TEC/10CV@AC	0/6 ^a^	0/6 ^a^
PLA/TEC/15CV@AC	0/6 ^a^	0/6 ^a^

^a^ 0/6: growth in the contact area in 6 out all six replicates, indicating strong antimicrobial activity.

**Table 8:** Odor, color, and texture scores of wrapped pork fillets during 10 days of storage at 4 ± 1 °C of minced pork wrapped in PLA/TEC and PLA/TEC/10CV@AC films as well as with a commercial film (control sample).

Sample	DAYS					
	0	2	4	6	8	10
	odor					
CONTROL	5.00 ± 0.00 ^a^	4.45 ± 0.10 ^b^	3.90 ± 0.20 ^d^	3.20 ± 0.10 ^g^	2.85 ± 0.10 ^j^	2.50 ± 0.10 ^m^
PLA/TEC	5.00 ± 0.00 ^a^	4.55 ± 0.10 ^b^	4.25 ± 0.10 ^e^	3.60 ± 0.20 ^h^	3.25 ± 0.18 ^k^	2.80 ± 0.20 ^m^
PLA/TEC/10CV@AC	5.00 ± 0.00 ^a^	4.68 ± 0.03 ^c,b^	4.51 ± 0.09 ^f^	4.12 ± 0.19 ^i^	3.91 ± 0.07 ^l^	3.16 ± 0.19 ^n,m^
	color					
CONTROL	5.00 ± 0.00 ^a^	4.30 ± 0.20 ^b^	3.98 ± 0.29 ^d^	2.85 ± 0.15 ^e^	2.70 ± 0.15 ^h^	2.55 ± 0.12 ^j^
PLA/TEC	5.00 ± 0.00 ^a^	4.50 ± 0.10 ^b^	4.35 ± 0.27 ^d^	3.45 ± 0.16 ^f^	2.85 ± 0.11 ^h^	2.70 ± 0.16 ^j^
PLA/TEC/10CV@AC	5.00 ± 0.00 ^a^	4.69 ± 0.05 ^c^	4.48 ± 0.21 ^d^	4.19 ± 0.12 ^g^	3.78 ± 0.06 ^i^	3.44 ± 0.11 ^k^
	texture					
CONTROL	5.00 ± 0.00 ^a^	4.48 ± 0.29 ^b^	3.80 ± 0.20 ^c^	3.32 ± 0.11 ^e^	2.84 ± 0.08 ^g^	2.54 ± 0.10 ^j^
PLA/TEC	5.00 ± 0.00 ^a^	4.40 ± 0.20 ^b^	4.22 ± 0.15 ^d^	3.70 ± 0.10 ^f^	3.22 ± 0.18 ^h^	2.75 ± 0.10 ^k^
PLA/TEC/10CV@AC	5.00 ± 0.00 ^a^	4.72 ± 0.18 ^b^	4.44 ± 0.15 ^d^	3.94 ± 0.18 ^f^	3.64 ± 0.16 ^i^	2.98 ± 0.21 ^k^

Different letters in each column indicate statistically significant differences at a confidence level of *p* < 0.05 (see also Figures S10–S12 and Tables S8–S10).

## Data Availability

Data are contained within the article and Supplementary Materials.

## Supplementary Materials

The following supporting information can be downloaded at: https://www.mdpi.com/article/10.3390/polym17050605/s1, Figure S1: Independent sample median test for E, σ, and %ε; Figure S2: Independent sample median test for D_wv_ and Pe_O2_; Figure S3: Equations for the determination of EC_50_ in PLA/TEC/5CV@AC films; Figure S4: Equations for the determination of EC_50_ in PLA/TEC/10CV@AC films; Figure S5: Equations for the determination of EC_50_ in PLA/TEC/15CV@AC films; Figure S6: Independent sample median test of EC_50_ values; Figure S7: Independent sample median test of TVC during storage time; Figure S8: Independent sample median test of TBARS during storage time; Figure S9: Independent sample median test of heme iron content during storage time; Figure S10: Independent sample median test of odor during storage time; Figure S11: Independent sample median test of color during storage time; Figure S12: Independent sample median test of texture during storage time; Table S1: Pairwise comparisons of the different treatments according to the mean values of E, σ, and %ε for all PLA/TEC/xAC and PLA/TEC/xCV@AC films; Table S2: WVTR, Dwv, O.T.R., and PeO_2_ values for all PLA/TEC/xAC and PLA/TEC/xCV@AC films as well as for pure PLA/TEC films; Table S3: Independent sample median test summary of the different treatments according to the mean values of EC_50_; Table S4: Pairwise comparisons of the different treatments according to the mean values of TVC during storage time; Table S5: Pairwise comparisons of the different treatments according to the mean values of TBARS during storage time; Table S6: Pairwise comparisons of the different treatments according to the mean values of heme iron content during storage time; Table S7: Pearson’s correlation between TBARS and heme iron content during storage time; Table S8: Pairwise comparisons of the different treatments according to the mean values of odor during storage time; Table S9: Pairwise comparisons of the different treatments according to the mean values of color during storage time; Table S10: Pairwise comparisons of the different treatments according to the mean values of texture during storage time; Video S1: Self-healing test of PLA/TEC/10CV@AC active film.
