# Supplementary material for: Novel Carvacrol@activated Carbon Nanohybrid for Innovative Poly(lactide Acid)/Triethyl Citrate Based Sustainable Active Packaging Films
Source: Polymers (Basel). 2025 Feb 24;17(5):605. doi: 10.3390/polym17050605 (PMC11902435; doi:10.3390/polym17050605)
Supplement: Supplementary file 1 [file polymers-17-00605-s001.zip › polymers-3483770-supplementary.pdf]

Supplementary material for

Carvacrol@Activated Carbon bioactive nanohybrids for poly-lactide acid/triethyl citrate based self-healable active packaging films applied in minced meat shelf-life extension

Vassilios K. Karabagias [1], Aris E. Giannakas [1,*], Areti A. Leontiou [1], Andreas Karydis-Messinis [2], Dimitrios Moschovas [2], Nikolaos D. Andritsos [1], Apostolos Avgeropoulos [2], Nikolaos E. Zafeiropoulos [2], Charalampos Proestos [3] and Constantinos E. Salmas [2,*]

[1] Department of Food Science and Technology, University of Patras, 30100 Agrinio, Greece; vkarampagias@upatras.gr (V.K.K.); aleontiu@upatras.gr (A.A.L.); nandritsos@upatras.gr (N.D.A.)
[2] Department of Material Science and Engineering, University of Ioannina, 45110 Ioannina, Greece; karydis.and@gmail.com (A.K.-M.); dmoschov@uoi.gr (D.M.); aavger@uoi.gr (A.A.); nzafirop@uoi.gr (N.E.Z.)
[3] Laboratory of Food Chemistry, Department of Chemistry, National and Kapodistrian University of Athens Zografou, 15771 Athens, Greece; harpro@chem.uoa.gr
* Correspondence: agiannakas@upatras.gr (A.E.G.); ksalmas@uoi.gr (C.E.S.)

2.4.1 CV desorption release kinetics of CV@AC

Approximately 100 mg of CV@AC nanohybrid was spread in the inner disk of moisture analyzer and its mass loss (m_t) was recorded as a function of time (t) at 50, 70, 90, and 110 °K. At least three desorption experiments were done at each temperature. From the recorded m_t and t values the desorption isotherms plots were constructed by plotting the values of $(1 - m_0/m_t)$ as a function of t. The plots were fitted using the well-known pseudo-second-order adsorption-desorption equation [67,68]. For process order, n=2 the overall normalized mass balance is given by:

$$\frac{dq_t}{dt} = k_2 * (q_e - q_t)^2 \quad \text{(S1)}$$

where k_2 is the rate constant of the pseudo-second-order kinetic model (s^{-1}), qt is the desorbed fraction capacity at time t, $q_e=(1-m_e/m_0)$ is the maximum desorbed fraction capacity at equilibrium, m_0 is the initial CV loading into the nanohybrid, and m_t is the CV amount remaining in the nanohybrid at time t. By integrating equation (1) we achieve the pseudo-second-order kinetic model:

$$q_t = (1 - \frac{m_t}{m_0}) = \frac{q_e^2 * k_2 * t}{q_e * k_2 * t + 1} \quad \text{(S2)}$$

The initial release rate can be computed via the equation (1) and for t=0 (i.e., q_t=0). Thus:

$$r_i = \left.\frac{dq_t}{dt}\right|_{t=0} = k_2 * q_e^2 \quad \text{(S3)}$$

From the best-fitted plots, the k_2 and q_e values were calculated. Using the estimated k_2 parameter the $\ln(k_2)$ term was calculated and plotted as a function of (1/T) to determine the desorption energy (E^0_{des}) according to the Arrhenius equation and the theory:

$$k_2 = k_0 \cdot e^{-\frac{E^0_{des}}{R \cdot T}} \quad \text{(S4)}$$

and its linear transformed type:

$$ln(k_2) = ln(k_0) - \frac{E^0_{des}}{R \cdot T} \quad \text{(S5)}$$

where k_2 is the rate constant of the pseudo-second order kinetic model (s^{-1}), E^0_{des} is the desorption activation energy, and A is the Arrhenius constant.

2.5.4 Mechanical and thermomechanical properties of films

At least three to five dog bone-shaped samples of all tested films were tensioned, and the stress–strain values were recorded. By using the applicable software (TrapeziumX version 1.5.6, Simantzü, Asteriadis, S.A., Athens, Greece) the elastic modulus E (MPa), ultimate strength σ_{uts} (MPa), and %elongation at break ε% Mean values were calculated.

All obtained PLA/TEC/xAC and PLA/TEC/xCV@AC films as well as pure PLA/TEC film were examined for their dynamic mechanical behaviors by using a dynamic mechanical analyzer (DMA Q800, TA Instruments, 159 Lukens Drive New Castle, DE, USA) in film tension mode. To evaluate the storage modulus (E), a temperature range of −20 °C to 60 °C at a rate of 5 K/min, along with a frequency of 1 Hz, was applied.

2.6.1 Water/oxygen barrier properties of films

Water barrier properties were determined at 38 °C and 95% RH according to the ASTM E96/E 96M-05 method and a handmade apparatus. For each film at least three to five samples were measured. The obtained Water Vapor Transmission Rate (WVTR) values were transformed to water vapor diffusion coefficient values (D_{wv}) according to the Fick's low theory, and the following equation:

$$D_{WV} = WVTR \cdot \frac{Dx}{DC} \quad \text{(S6)}$$

where WVTR [g/(cm^2.s)] is the water vapor transmission rate, Dx (cm) is the film thickness, and DC (g/cm^3) is the humidity concentration gradient on the two opposite sides of the film.
The oxygen transmission rate (OTR) values of all PLA/TEC/xAC and PLA/TEC/xCV@AC films as well as PLA/TEC film were determined according to ASTM D 3985 method at 23 °C and 0% RH using an oxygen permeation analyzer (O.P.A., 8001, Systech Illinois Instruments Co., Johnsburg, IL, USA). At least three to five samples of each film were measured. Average thickness (Δx) of each film was the average value of thickness values in twelve different points of film. The obtained OTR (ml/m^2/day) values were transformed to oxygen permeability (Pe_{O2} – cm/s) values using the methodology described in detail recently and the following equation:

$$Pe_{O2} = 10^{-5}.\Delta x.OTR/86400 \quad \text{(S7)}$$

2.6.2 Desorption kinetics of PLA/TEC/xCV@AC films

For each film three to five samples 900 to 1000 mg were placed inside the moisture analyzer, and the mass loss values were recorded as function of time at 70 °C for 1 h. Using the obtained film mass loss values (m_t) as a function of time (t), for each film, the CV desorption kinetic plots were constructed by plotting the values of 1-m_t/m_0 as a function of time. These plots were fitted with pseudo second order kinetic model to obtain k_2, and q_e mean values for all PLA/TEC/xCV@AC active films.

2.6.3 Antioxidant activity of PLA/TEC/xCV@AC films

A DPPH radical methanolic standard solution of 2.16 mM (mmol/L) was prepared and used for all experiments. Dilutions were made to obtain 10, 20, 30, 40, and 50 mg/L DPPH solutions. For the preparation of calibration curve the absorbance of these five solutions was

measured at 517 nm using a SHIMADZU UV-1900 UV/VIS Spectrometer. The calibration curve of absorbance (y) versus the concentration (x) of [DPPH•] free radical was found to follow the next equation:

$$y = 0.0388x + 0.015; R^2 = 0.9994 \quad (S8)$$

Next, the determination of the concentration required to obtain a 50% antioxidant effect (EC_{50}) from all obtained PLA/TEC/xCV@AC active films was done. 10, 20, 30, 40, and 50 mg of granule film were placed in dark vials in triplicates and 3 mL of standard DPPH radical methanolic solution and 2 mL of acetate buffer 100 mM (pH = 7.10) were added to each vial. All prepared dark vials were kept under dark conditions for 8 hours and next the absorbance was measured at 517 nm. As a blank sample, a vial containing 3 mL of standard DPPH radical methanolic solution and 2 mL of acetate buffer without the addition of any granule film was used. The % inhibition of DPPH radical was calculated using the following equation:

$$\% \text{ scavenged DPPH}^{\bullet} \text{ at steady state } = \frac{A_0^{517} - A_{\text{sample}}^{517}}{A_0^{517}} \times 100 \quad (S9)$$

2.6.4 Antibacterial activity of PLA/TEC/xCV@AC films

The antimicrobial activity of all obtained PLA/TEC/xCV@AC active films was tested against one Gram-positive *Staphylococcus aureus* (NCTC 6571) and one Gram-negative *Salmonella enterica* subspecies *enterica* serovar Typhimurium (NCTC 12023) food pathogens.

The experimental procedure followed was based on the agar diffusion method described in detail recently [37]. The diameters of inhibition zones in the contact area of the films and around them were measured using a Vernier caliper at 0.1 mm accuracy. The experimental procedure was repeated twice while films were measured in triplicate in each repetition. The bacteria *Staphylococcus aureus* (NCTC 6571) and *Salmonella enterica subspecies enterica serovar Typhimurium* (NCTC 12023) were supplied by Supelco® Analytical Products, a subsidiary of Merck (Darmstadt, Germany) as microbiological certified reference materials in the form of easy-tab™ pellets (S. aureus) by LGC Standards Proficiency Testing (Chamberhall Green Bury, Lancashire, UK) and in the form of disc-shaped Vitroids™ (S. Typhimurium). one Gram-negative

2.7.1. Packaging Preservation Test of Minced Pork Meat

Minced pork, in portions of approximately 70–80 g each, were aseptically wrapped between two films of PLA/TEC and PLA/TEC10CV@AC samples that were 11 cm in diameter and placed inside the Aifantis company's commercial wrapping paper without the inner film (coated with plasticized PVC). As a control sample, 80–90 g of minced pork was aseptically wrapped in the commercial opaque packaging paper (without removing the inner coated PVC film) from the Aifantis company. For all tested packaging systems, samples for the 2nd , 4th , 6th , 8th , and 10th day of preservation were prepared and stored at a temperature of 4 ± 1 °C (LG GC-151SA,Weybridge, UK).

2.7.2. Lipid Oxidation of Minced Pork Meat with Thiobarbituric Acid Reactive Substances

Lipid oxidation rates of all minced pork samples during the 10 days of storage were determined by following the thiobarbituric acid reactive substances (TBARS) method according to the methodology described in detail recently [37].

TBARS was expressed as mg of malondialdehyde (MDA)/kg of the sample using the following equation:

$$\text{TBARS (mg/kg)} = 3.6 \times D \quad (S10)$$

2.7.3. *Heme* Iron Content

The heme iron content values of all minced pork samples during the 10 days of storage were determined according to the methodology described in detail recently [37]. The heme iron content in minced pork was calculated using the equation (11):

$$\text{Heme iron } (\mu g/g) = A_{640} \times 680 \times 0.0882 \qquad \text{(S11)}$$

where A_{640} is the absorbance measured at 640 nm and 680 and 0.0882 are constant values in the equation.

2.7.4. Total Viable Count (TVC) of Minced Pork Meat

For the estimation of total viable count (TVC) for all minced pork samples during the 10 days of storage, we used the methodology described in detail recently [37].

2.7.5. Sensory Analysis of Minced Pork Meat

Color, odor, and texture of wrapped minced pork were evaluated during the 10 days of storage. All the above properties were ranked from 0 (lowest degree of each characteristic in the tested samples) to 5 (highest degree of each characteristic in the tested samples) for each packaging treatment, at each sampling day (2nd, 4th, 6th, 8th, and 10th day of storage) by seven panelist/members of the Department of Food Science and Technology experienced in meat sensory evaluation using the conventional descriptive analysis [74–76]. A descriptive analysis panel determined the important product characteristics and then evaluated the degree of each characteristic in the tested samples.

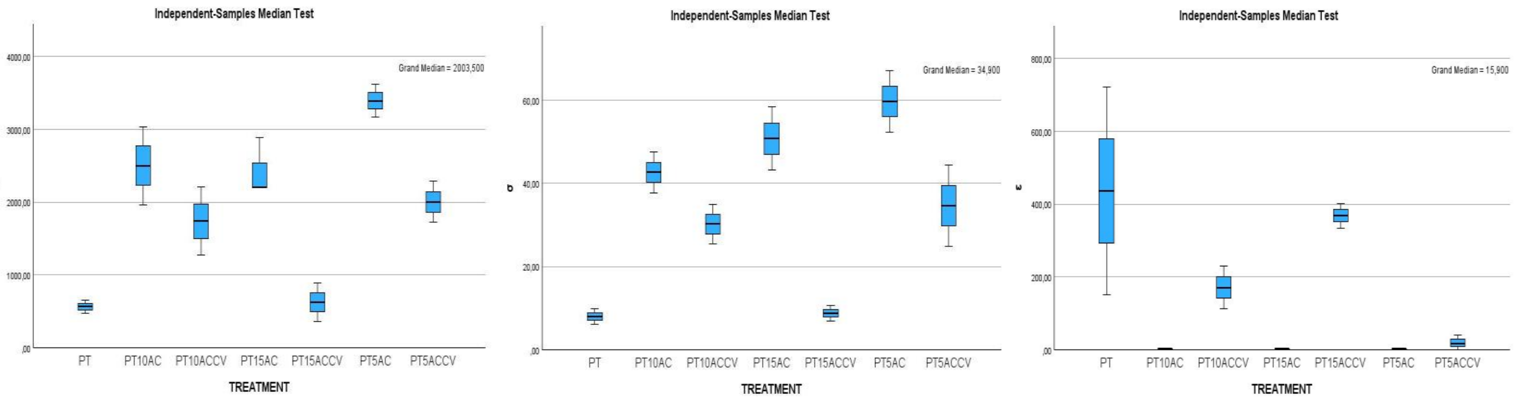

Figure S1. Independent-Samples Median Test for E, σ, %ε

Table S1. Pairwise Comparisons of the different treatments according to the mean values of E, σ, %ε.

E				σ				%ε			
**Pairwise Comparisons of TREATMENT**				**Pairwise Comparisons of TREATMENT**				**Pairwise Comparisons of TREATMENT**			
Sample 1-Sample 2	Test Statistic	Sig.	Adj. Sig.[a]	Sample 1-Sample 2	Test Statistic	Sig.	Adj. Sig.[a]	Sample 1-Sample 2	Test Statistic	Sig.	Adj. Sig.[a]
PT-PT15ACCV	0,667	0,414	1,000	PT-PT15ACCV	0,667	0,414	1,000	PT10AC-PT15AC	0,667	0,414	1,000
PT-PT10ACCV	6,000	0,014	0,300	PT-PT10ACCV	6,000	0,014	0,300	PT10AC-PT5AC	0,667	0,414	1,000
PT-PT5ACCV	6,000	0,014	0,300	PT-PT5ACCV	6,000	0,014	0,300	PT10AC-PT5ACCV	0,667	0,414	1,000
PT-PT15AC	6,000	0,014	0,300	PT-PT10AC	6,000	0,014	0,300	PT10AC-PT10ACCV	6,000	0,014	0,300
PT-PT10AC	6,000	0,014	0,300	PT-PT15AC	6,000	0,014	0,300	PT10AC-PT15ACCV	6,000	0,014	0,300

PT-PT5AC	6,000	0,014	0,300	PT-PT5AC	6,000	0,014	0,300	PT10AC-PT	6,000	0,014	0,300
PT15ACCV-PT10ACCV	6,000	0,014	0,300	PT15ACCV-PT10ACCV	6,000	0,014	0,300	PT15AC-PT5AC	0,667	0,414	1,000
PT15ACCV-PT5ACCV	6,000	0,014	0,300	PT15ACCV-PT5ACCV	6,000	0,014	0,300	PT15AC-PT5ACCV	0,667	0,414	1,000
PT15ACCV-PT15AC	6,000	0,014	0,300	PT15ACCV-PT10AC	6,000	0,014	0,300	PT15AC-PT10ACCV	6,000	0,014	0,300
PT15ACCV-PT10AC	6,000	0,014	0,300	PT15ACCV-PT15AC	6,000	0,014	0,300	PT15AC-PT15ACCV	6,000	0,014	0,300
PT15ACCV-PT5AC	6,000	0,014	0,300	PT15ACCV-PT5AC	6,000	0,014	0,300	PT15AC-PT	6,000	0,014	0,300
PT10ACCV-PT5ACCV	0,667	0,414	1,000	PT10ACCV-PT5ACCV	0,667	0,414	1,000	PT5AC-PT5ACCV	0,667	0,414	1,000
PT10ACCV-PT15AC	0,000	1,000	1,000	PT10ACCV-PT10AC	6,000	0,014	0,300	PT5AC-PT10ACCV	6,000	0,014	0,300
PT10ACCV-PT10AC	0,667	0,414	1,000	PT10ACCV-PT15AC	6,000	0,014	0,300	PT5AC-PT15ACCV	6,000	0,014	0,300
PT10ACCV-PT5AC	6,000	0,014	0,300	PT10ACCV-PT5AC	6,000	0,014	0,300	PT5AC-PT	6,000	0,014	0,300
PT5ACCV-PT15AC	0,000	1,000	1,000	PT5ACCV-PT10AC	0,667	0,414	1,000	PT5ACCV-PT10ACCV	6,000	0,014	0,300
PT5ACCV-PT10AC	0,667	0,414	1,000	PT5ACCV-PT15AC	0,667	0,414	1,000	PT5ACCV-PT15ACCV	6,000	0,014	0,300
PT5ACCV-PT5AC	6,000	0,014	0,300	PT5ACCV-PT5AC	6,000	0,014	0,300	PT5ACCV-PT	6,000	0,014	0,300
PT15AC-PT10AC	0,667	0,414	1,000	PT10AC-PT15AC	0,667	0,414	1,000	PT10ACCV-PT15ACCV	6,000	0,014	0,300
PT15AC-PT5AC	6,000	0,014	0,300	PT10AC-PT5AC	6,000	0,014	0,300	PT10ACCV-PT	0,667	0,414	1,000
PT10AC-PT5AC	6,000	0,014	0,300	PT15AC-PT5AC	0,667	0,414	1,000	PT15ACCV-PT	0,667	0,414	1,000
Each row tests the null hypothesis that the Sample 1 and Sample 2 distributions are the same. Asymptotic significances (2-sided tests) are displayed. The significance level is ,050.				Each row tests the null hypothesis that the Sample 1 and Sample 2 distributions are the same. Asymptotic significances (2-sided tests) are displayed. The significance level is ,050.				Each row tests the null hypothesis that the Sample 1 and Sample 2 distributions are the same. Asymptotic significances (2-sided tests) are displayed. The significance level is ,050.			
a. Significance values have been adjusted by the Bonferroni correction for multiple tests.				a. Significance values have been adjusted by the Bonferroni correction for multiple tests.				a. Significance values have been adjusted by the Bonferroni correction for multiple tests.			

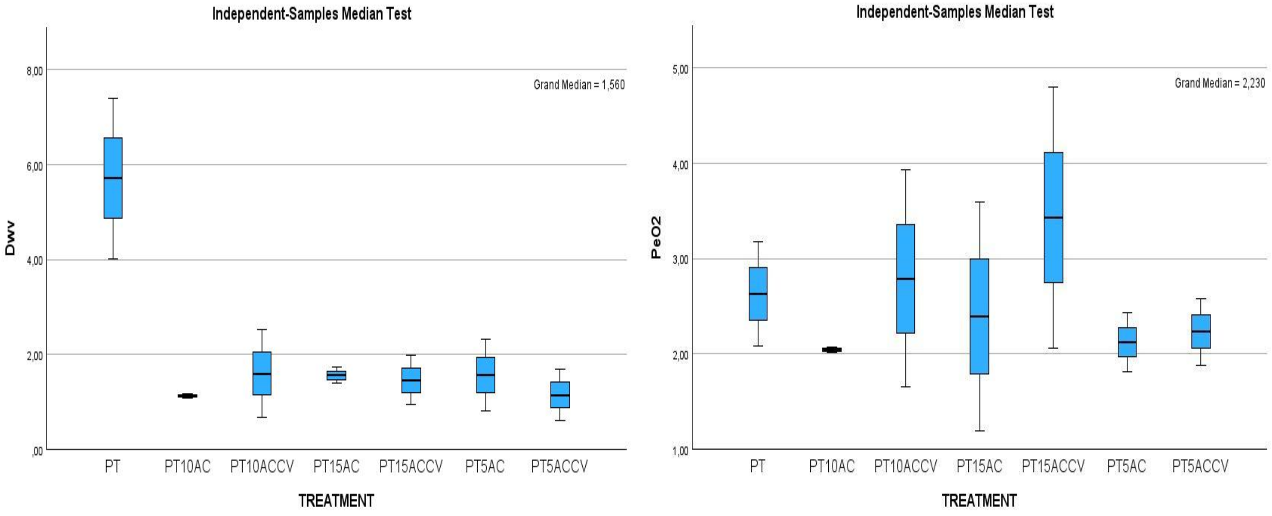

Figure S2. Independent-Samples Median Test for Dwv,PeO_2.

Table S2. Pairwise Comparisons of the different treatments according to the mean values of Dwv,PeO2.

**Dwv**				**PeO2**			
Pairwise Comparisons of TREATMENT				Pairwise Comparisons of TREATMENT			
Sample 1-Sample 2	Test Statistic	Sig.	Adj. Sig.[a]	Sample 1-Sample 2	Test Statistic	Sig.	Adj. Sig.[a]
PT10AC-PT5ACCV	0,667	0,414	1,000	PT10AC-PT5AC	0,667	0,414	1,000
PT10AC-PT15ACCV	0,667	0,414	1,000	PT10AC-PT5ACCV	0,667	0,414	1,000
PT10AC-PT15AC	6,000	0,014	0,300	PT10AC-PT15AC	0,667	0,414	1,000
PT10AC-PT5AC	0,667	0,414	1,000	PT10AC-PT	6,000	0,014	0,300
PT10AC-PT10ACCV	0,667	0,414	1,000	PT10AC-PT10ACCV	0,667	0,414	1,000
PT10AC-PT	6,000	0,014	0,300	PT10AC-PT15ACCV	0,667	0,414	1,000
PT5ACCV-PT15ACCV	0,667	0,414	1,000	PT5AC-PT5ACCV	0,667	0,414	1,000
PT5ACCV-PT15AC	0,667	0,414	1,000	PT5AC-PT15AC	0,667	0,414	1,000
PT5ACCV-PT5AC	0,667	0,414	1,000	PT5AC-PT	0,667	0,414	1,000
PT5ACCV-PT10ACCV	0,667	0,414	1,000	PT5AC-PT10ACCV	0,667	0,414	1,000
PT5ACCV-PT	6,000	0,014	0,300	PT5AC-PT15ACCV	0,667	0,414	1,000
PT15ACCV-PT15AC	0,667	0,414	1,000	PT5ACCV-PT15AC	0,667	0,414	1,000
PT15ACCV-PT5AC	0,667	0,414	1,000	PT5ACCV-PT	0,667	0,414	1,000

PT15ACCV-PT10ACCV	0,667	0,414	1,000	PT5ACCV-PT10ACCV	0,667	0,414	1,000
PT15ACCV-PT	6,000	0,014	0,300	PT5ACCV-PT15ACCV	0,667	0,414	1,000
PT15AC-PT5AC	0,667	0,414	1,000	PT15AC-PT	0,667	0,414	1,000
PT15AC-PT10ACCV	0,667	0,414	1,000	PT15AC-PT10ACCV	0,667	0,414	1,000
PT15AC-PT	6,000	0,014	0,300	PT15AC-PT15ACCV	0,667	0,414	1,000
PT5AC-PT10ACCV	0,667	0,414	1,000	PT-PT10ACCV	0,667	0,414	1,000
PT5AC-PT	6,000	0,014	0,300	PT-PT15ACCV	0,667	0,414	1,000
PT10ACCV-PT	6,000	0,014	0,300	PT10ACCV-PT15ACCV	0,667	0,414	1,000
Each row tests the null hypothesis that the Sample 1 and Sample 2 distributions are the same. Asymptotic significances (2-sided tests) are displayed. The significance level is ,050.				Each row tests the null hypothesis that the Sample 1 and Sample 2 distributions are the same. Asymptotic significances (2-sided tests) are displayed. The significance level is ,050.			
a. Significance values have been adjusted by the Bonferroni correction for multiple tests.				a. Significance values have been adjusted by the Bonferroni correction for multiple tests.			

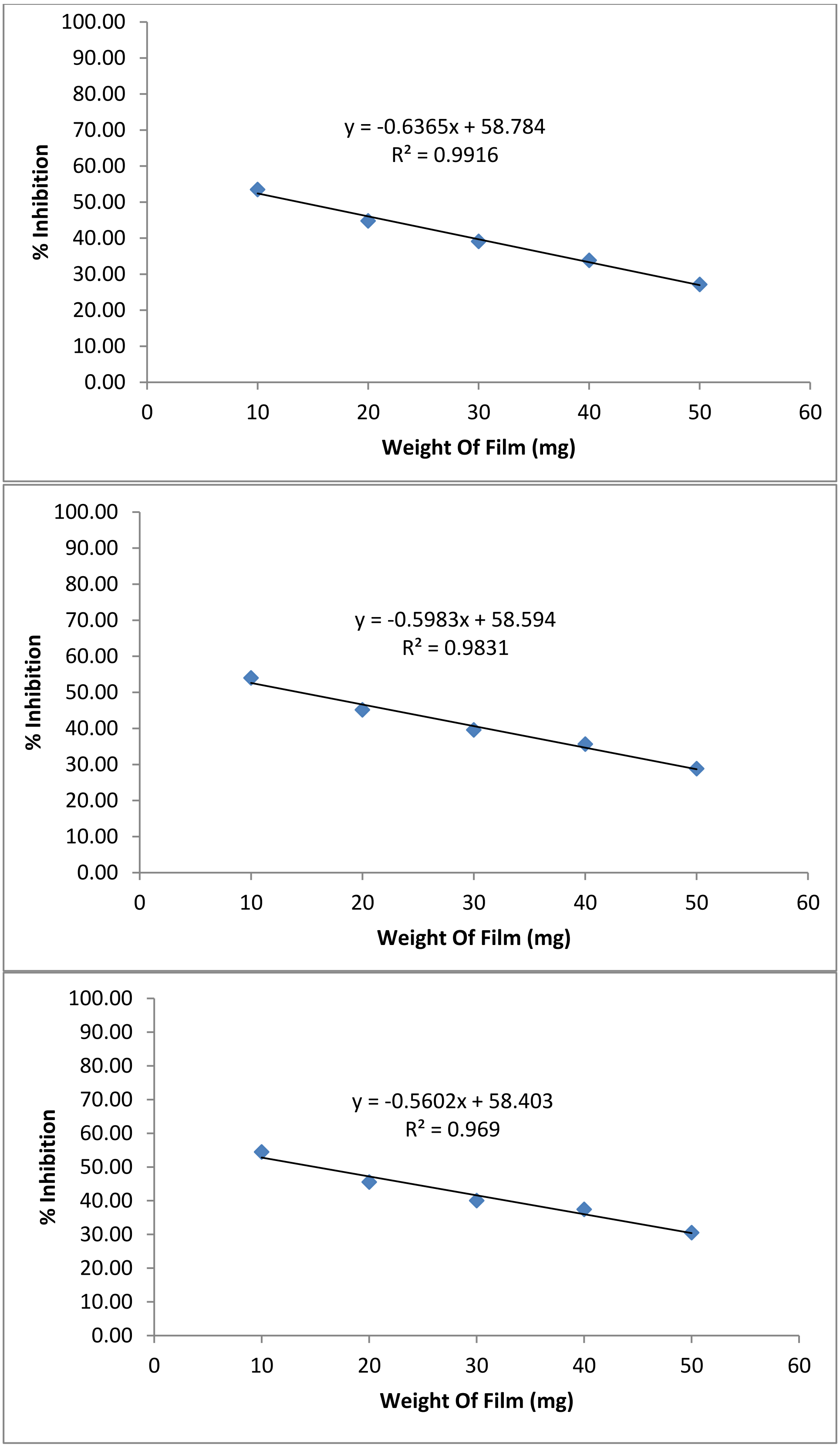

Figure S3. Equations for the determination of EC_{50} in PLA/TEC/5CV@AC films.

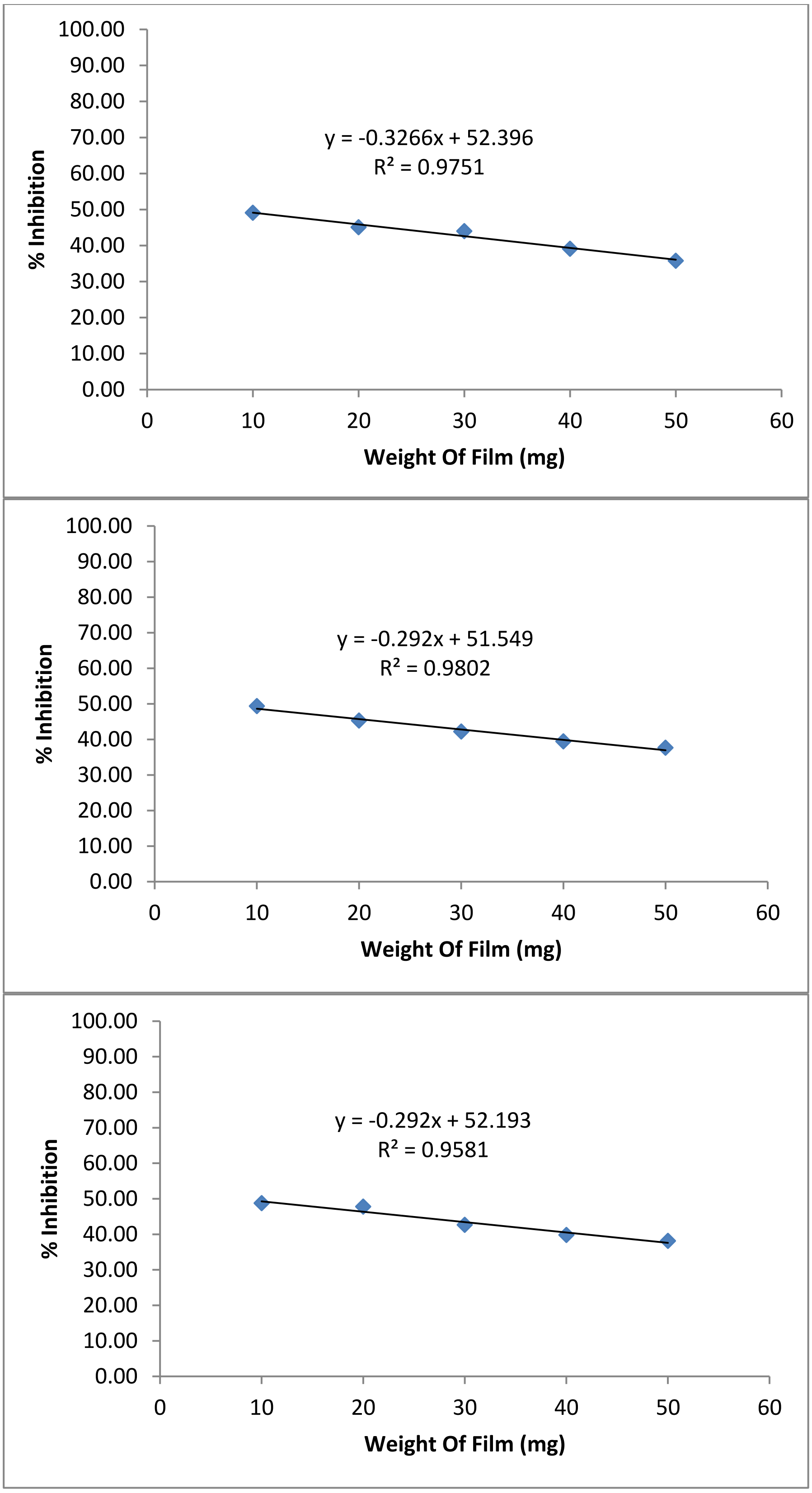

Figure S4. Equations for the determination of EC_{50} in PLA/TEC/10CV@AC films.

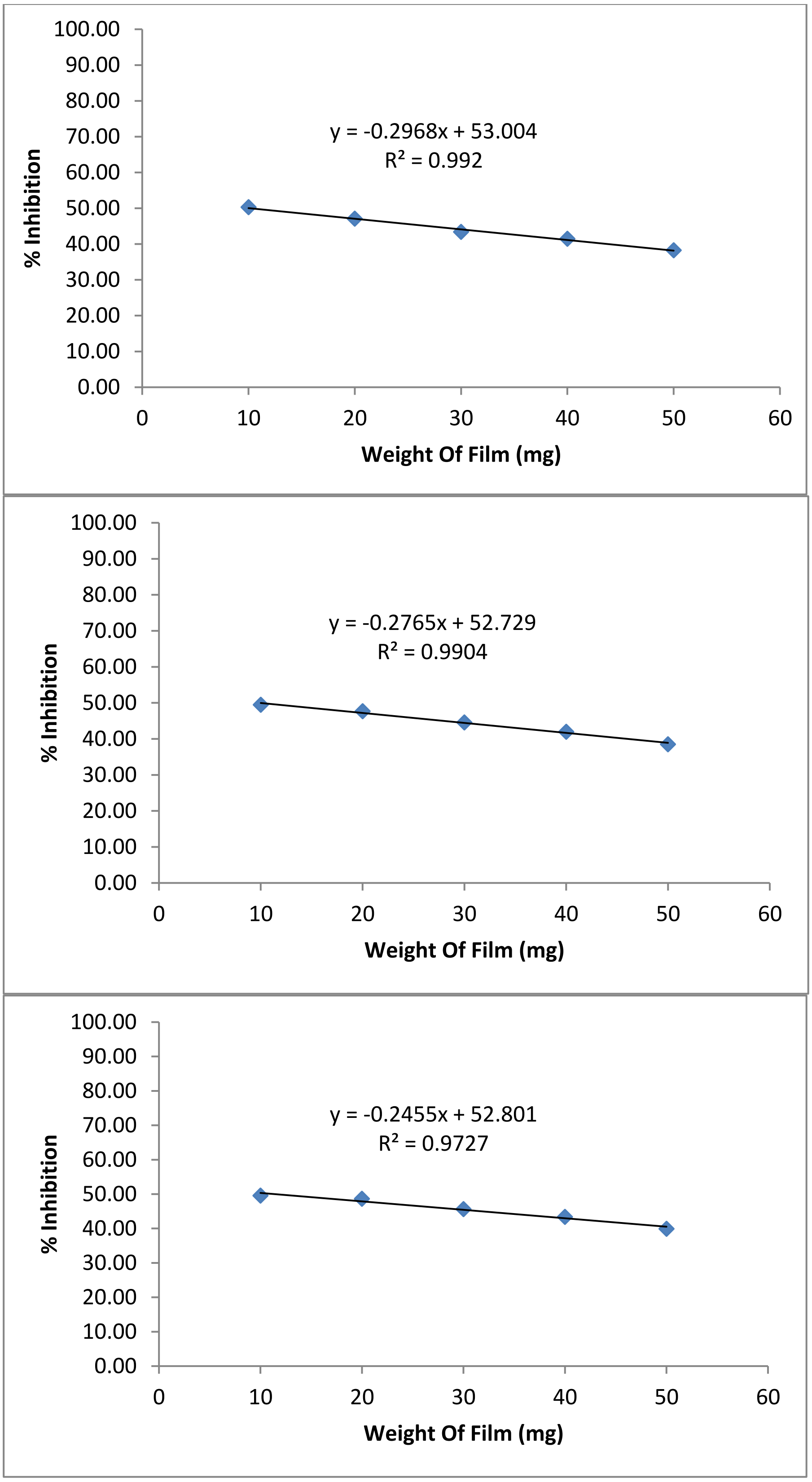

Figure S5. Equations for the determination of EC_{50} in PLA/TEC/15CV@AC films.

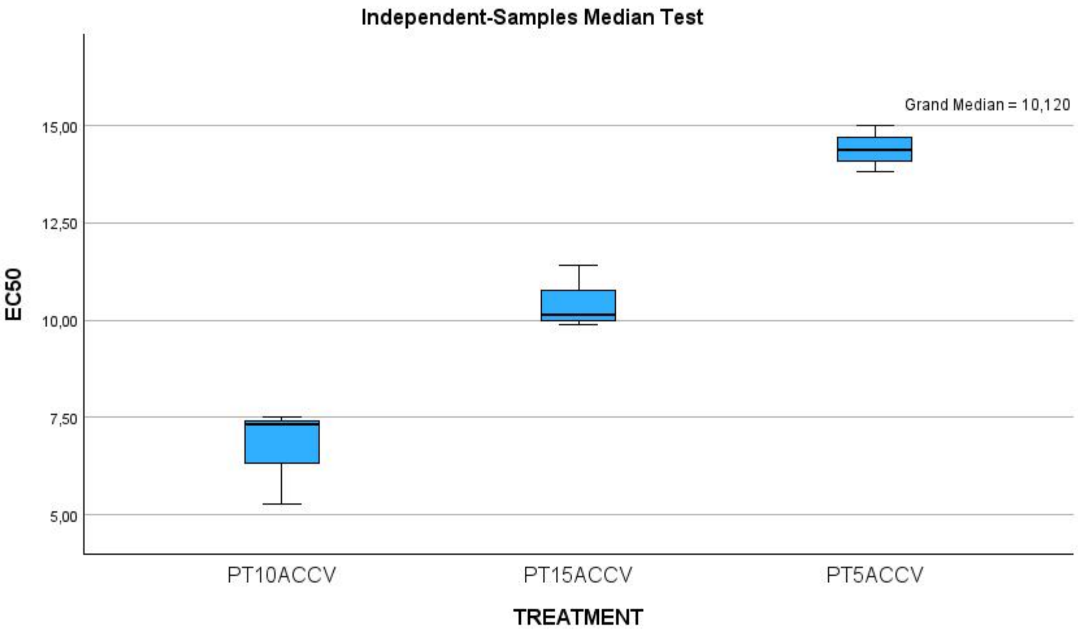

Figure S6. Independent-Samples Median Test of EC_{50} values.

Table S3. Independent-Samples Median Test Summary of the different treatments according to the mean values of EC_{50}

Pairwise Comparisons of TREATMENT			
Sample 1-Sample 2	Test Statistic	Sig.	Adj. Sig.a
PT10ACCV-PT15ACCV	6,000	0,014	0,043
PT10ACCV-PT5ACCV	6,000	0,014	0,043
PT15ACCV-PT5ACCV	6,000	0,014	0,043
Each row tests the null hypothesis that the Sample 1 and Sample 2 distributions are the same. Asymptotic significances (2-sided tests) are displayed. The significance level is ,050.			
a. Significance values have been adjusted by the Bonferroni correction for multiple tests.			

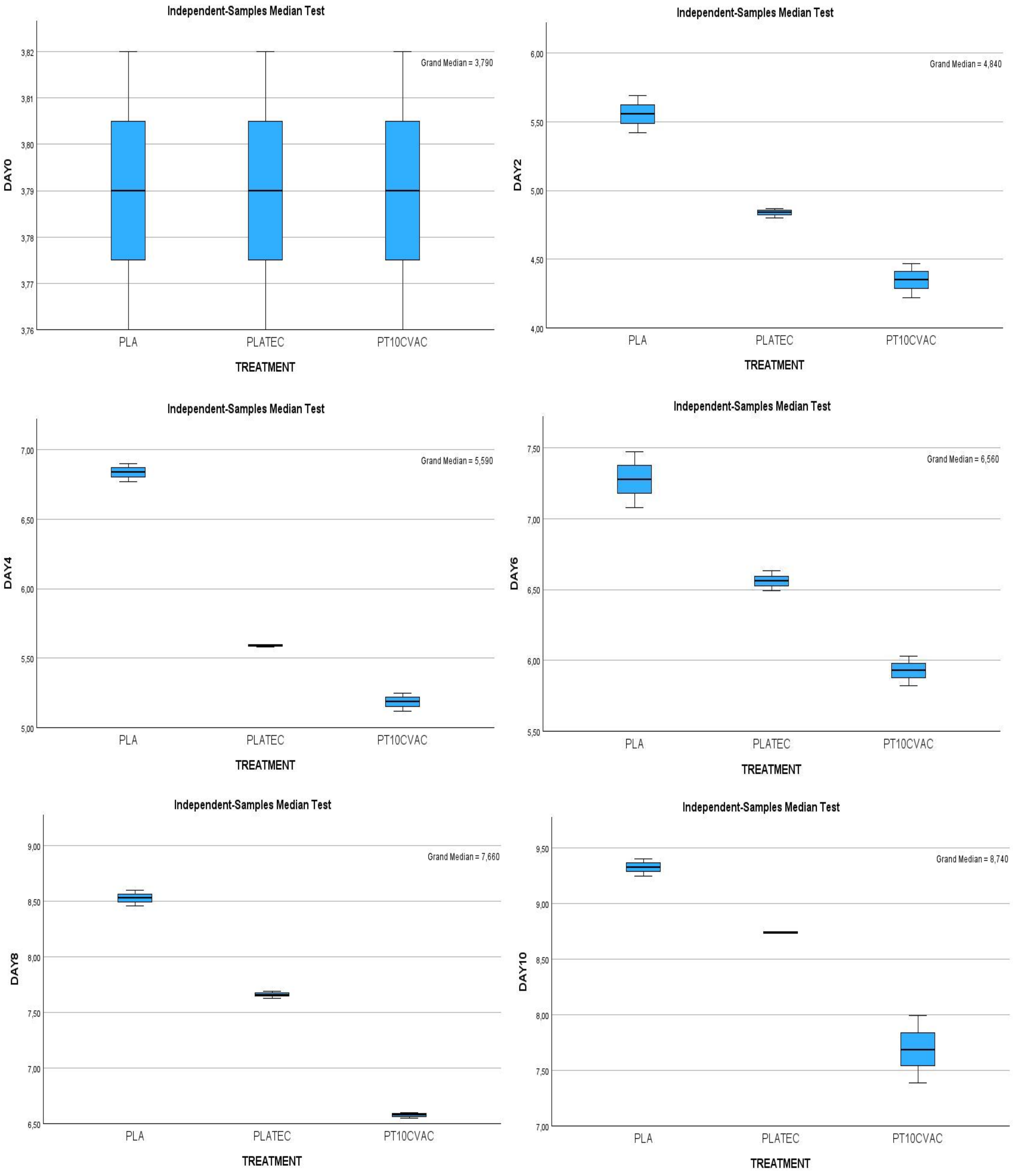

Figure S7. Independent-Samples Median Test of TVC durring storage time.

Table S4. Pairwise Comparisons of the different treatments according to the mean values of TVC during storage time.

DAY 0			
**Pairwise Comparisons of TREATMENT**			
Sample 1-Sample 2	Test Statistic	Sig.	Adj. Sig.[a]
PLA-PLATEC	0,000	1,000	1,000
PLA-PT10CVAC	0,000	1,000	1,000
PLATEC-PT10CVAC	0,000	1,000	1,000
Each row tests the null hypothesis that the Sample 1 and Sample 2 distributions are the same. Asymptotic significances (2-sided tests) are displayed. The significance level is ,050.			
a. Significance values have been adjusted by the Bonferroni correction for multiple tests.			

DAY 2			
**Pairwise Comparisons of TREATMENT**			
Sample 1-Sample 2	Test Statistic	Sig.	Adj. Sig.[a]
PT10CVAC-PLATEC	6,000	0,014	0,043
PT10CVAC-PLA	6,000	0,014	0,043
PLATEC-PLA	6,000	0,014	0,043
Each row tests the null hypothesis that the Sample 1 and Sample 2 distributions are the same. Asymptotic significances (2-sided tests) are displayed. The significance level is ,050.			
a. Significance values have been adjusted by the Bonferroni correction for multiple tests.			

DAY 4			
**Pairwise Comparisons of TREATMENT**			
Sample 1-Sample 2	Test Statistic	Sig.	Adj. Sig.[a]
PT10CVAC-PLATEC	6,000	0,014	0,043
PT10CVAC-PLA	6,000	0,014	0,043
PLATEC-PLA	6,000	0,014	0,043
Each row tests the null hypothesis that the Sample 1 and Sample 2 distributions are the same. Asymptotic significances (2-sided tests) are displayed. The significance level is ,050.			
a. Significance values have been adjusted by the Bonferroni correction for multiple tests.			

DAY 6			
**Pairwise Comparisons of TREATMENT**			
Sample 1-Sample 2	Test Statistic	Sig.	Adj. Sig.[a]
PT10CVAC-PLATEC	6,000	0,014	0,043
PT10CVAC-PLA	6,000	0,014	0,043
PLATEC-PLA	6,000	0,014	0,043
Each row tests the null hypothesis that the Sample 1 and Sample 2 distributions are the same. Asymptotic significances (2-sided tests) are displayed. The significance level is ,050.			
a. Significance values have been adjusted by the Bonferroni correction for multiple tests.			

DAY 8			
**Pairwise Comparisons of TREATMENT**			
Sample 1-Sample 2	Test Statistic	Sig.	Adj. Sig.[a]
PT10CVAC-PLATEC	6,000	0,014	0,043
PT10CVAC-PLA	6,000	0,014	0,043
PLATEC-PLA	6,000	0,014	0,043
Each row tests the null hypothesis that the Sample 1 and Sample 2 distributions are the same. Asymptotic significances (2-sided tests) are displayed. The significance level is ,050.			
a. Significance values have been adjusted by the Bonferroni correction for multiple tests.			

DAY 10			
**Pairwise Comparisons of TREATMENT**			
Sample 1-Sample 2	Test Statistic	Sig.	Adj. Sig.[a]
PT10CVAC-PLATEC	6,000	0,014	0,043
PT10CVAC-PLA	6,000	0,014	0,043
PLATEC-PLA	6,000	0,014	0,043
Each row tests the null hypothesis that the Sample 1 and Sample 2 distributions are the same. Asymptotic significances (2-sided tests) are displayed. The significance level is ,050.			
a. Significance values have been adjusted by the Bonferroni correction for multiple tests.			

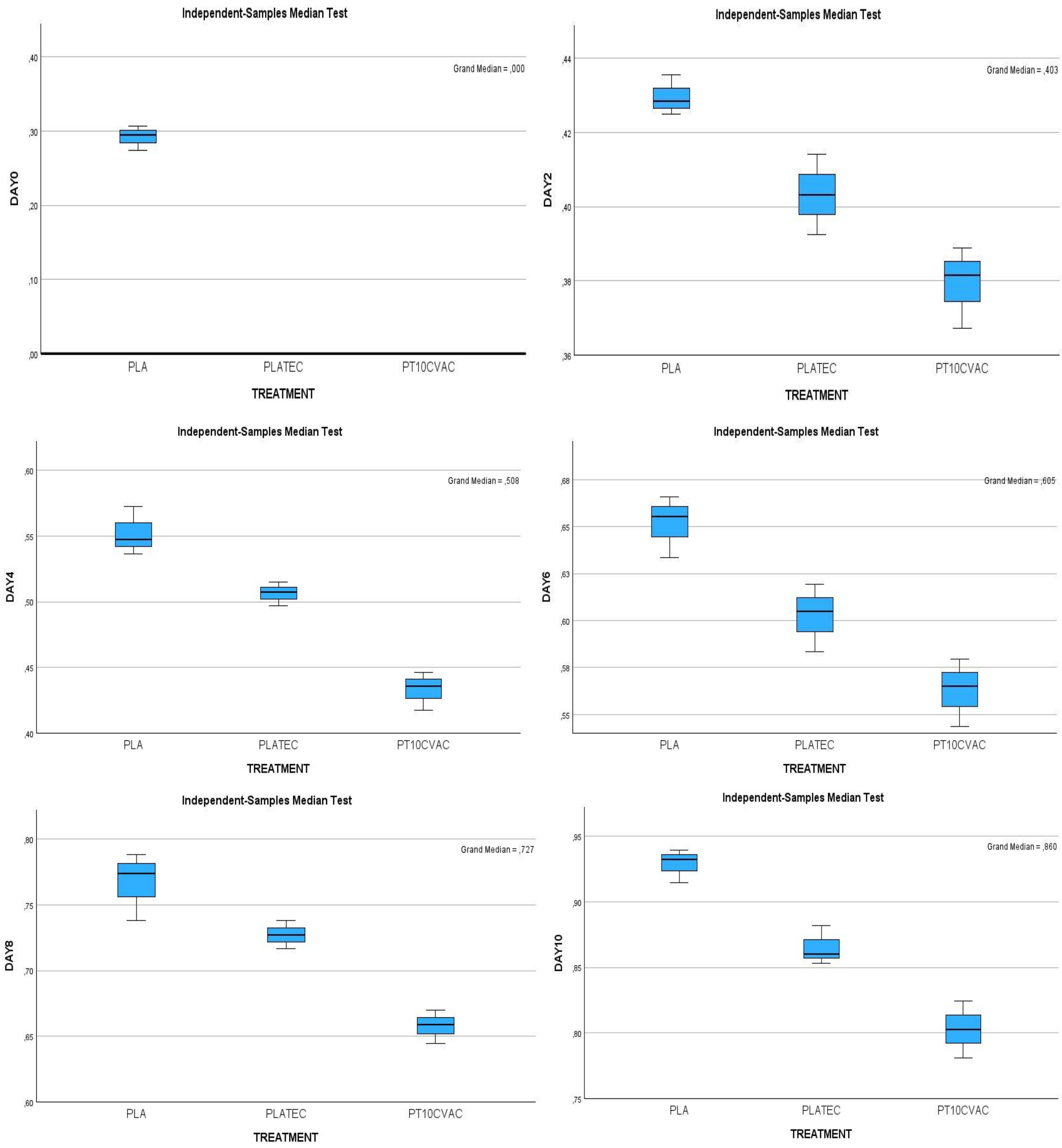

Figure S8. Independent-Samples Median Test of TBARS durring storage time.

Table S5. Pairwise Comparisons of the different treatments according to the mean values of TBARS during storage time.

DAY 0				DAY 2				DAY 4			
**Pairwise Comparisons of TREATMENT**				**Pairwise Comparisons of TREATMENT**				**Pairwise Comparisons of TREATMENT**			
Sample 1-Sample 2	Test Statistic	Sig.	Adj. Sig.[a]	Sample 1-Sample 2	Test Statistic	Sig.	Adj. Sig.[a]	Sample 1-Sample 2	Test Statistic	Sig.	Adj. Sig.[a]
PLATEC-PLA	6,000	0,014	0,043	PT10CVAC-PLATEC	6,000	0,014	0,043	PT10CVAC-PLATEC	6,000	0,014	0,043
PT10CVAC-PLA	6,000	0,014	0,043	PT10CVAC-PLA	6,000	0,014	0,043	PT10CVAC-PLA	6,000	0,014	0,043
PLATEC-PT10CVAC	.[b]			PLATEC-PLA	6,000	0,014	0,043	PLATEC-PLA	6,000	0,014	0,043
Each row tests the null hypothesis that the Sample 1 and Sample 2 distributions are the same. Asymptotic significances (2-sided tests) are displayed. The significance level is ,050.				Each row tests the null hypothesis that the Sample 1 and Sample 2 distributions are the same. Asymptotic significances (2-sided tests) are displayed. The significance level is ,050.				Each row tests the null hypothesis that the Sample 1 and Sample 2 distributions are the same. Asymptotic significances (2-sided tests) are displayed. The significance level is ,050.			
a. Significance values have been adjusted by the Bonferroni correction for multiple tests.				a. Significance values have been adjusted by the Bonferroni correction for multiple tests.				a. Significance values have been adjusted by the Bonferroni correction for multiple tests.			
b. Unable to compute because all sample medians in this pair are less than or equal to the hypothesized median.											
**DAY 6**				**DAY 8**				**DAY 10**			
**Pairwise Comparisons of TREATMENT**				**Pairwise Comparisons of TREATMENT**				**Pairwise Comparisons of TREATMENT**			
Sample 1-Sample 2	Test Statistic	Sig.	Adj. Sig.[a]	Sample 1-Sample 2	Test Statistic	Sig.	Adj. Sig.[a]	Sample 1-Sample 2	Test Statistic	Sig.	Adj. Sig.[a]
PT10CVAC-PLATEC	6,000	0,014	0,043	PT10CVAC-PLATEC	6,000	0,014	0,043	PT10CVAC-PLATEC	6,000	0,014	0,043
PT10CVAC-PLA	6,000	0,014	0,043	PT10CVAC-PLA	6,000	0,014	0,043	PT10CVAC-PLA	6,000	0,014	0,043
PLATEC-PLA	6,000	0,014	0,043	PLATEC-PLA	3,000	0,083	0,250	PLATEC-PLA	6,000	0,014	0,043
Each row tests the null hypothesis that the Sample 1 and Sample 2 distributions are the same. Asymptotic significances (2-sided tests) are displayed. The significance level is ,050.				Each row tests the null hypothesis that the Sample 1 and Sample 2 distributions are the same. Asymptotic significances (2-sided tests) are displayed. The significance level is ,050.				Each row tests the null hypothesis that the Sample 1 and Sample 2 distributions are the same. Asymptotic significances (2-sided tests) are displayed. The significance level is ,050.			
a. Significance values have been adjusted by the Bonferroni correction for multiple tests.				a. Significance values have been adjusted by the Bonferroni correction for multiple tests.				a. Significance values have been adjusted by the Bonferroni correction for multiple tests.			

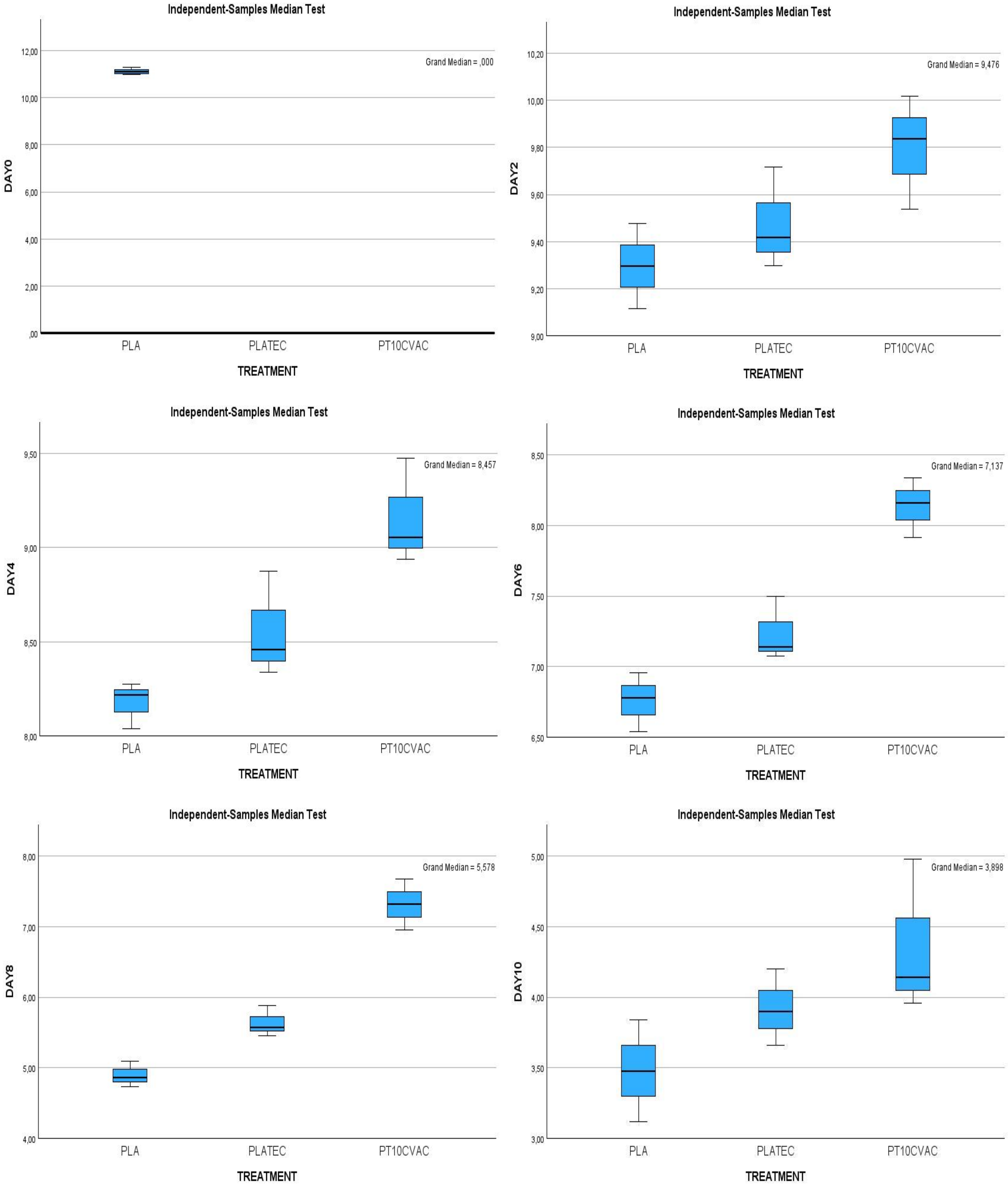

Figure S9. Independent-Samples Median Test of Heme-iron content durring storage time.

Table S6. Pairwise Comparisons of the different treatments according to the mean values of Heme-iron content during storage time.

**DAY 0**				**DAY 2**				**DAY 4**			
**Pairwise Comparisons of TREATMENT**				**Pairwise Comparisons of TREATMENT**				**Pairwise Comparisons of TREATMENT**			
Sample 1-Sample 2	Test Statistic	Sig.	Adj. Sig.[a]	Sample 1-Sample 2	Test Statistic	Sig.	Adj. Sig.[a]	Sample 1-Sample 2	Test Statistic	Sig.	Adj. Sig.[a]
PLATEC-PLA	6,000	0,014	0,043	PLA-PLATEC	0,667	0,414	1,000	PLA-PLATEC	6,000	0,014	0,043
PT10CVAC-PLA	6,000	0,014	0,043	PLA-PT10CVAC	6,000	0,014	0,043	PLA-PT10CVAC	6,000	0,014	0,043
PLATEC-PT10CVAC	.[b]			PLATEC-PT10CVAC	0,667	0,414	1,000	PLATEC-PT10CVAC	6,000	0,014	0,043
Each row tests the null hypothesis that the Sample 1 and Sample 2 distributions are the same. Asymptotic significances (2-sided tests) are displayed. The significance level is ,050.				Each row tests the null hypothesis that the Sample 1 and Sample 2 distributions are the same. Asymptotic significances (2-sided tests) are displayed. The significance level is ,050.				Each row tests the null hypothesis that the Sample 1 and Sample 2 distributions are the same. Asymptotic significances (2-sided tests) are displayed. The significance level is ,050.			
a. Significance values have been adjusted by the Bonferroni correction for multiple tests.				a. Significance values have been adjusted by the Bonferroni correction for multiple tests.				a. Significance values have been adjusted by the Bonferroni correction for multiple tests.			
b. Unable to compute because all sample medians in this pair are less than or equal to the hypothesized median.											
**DAY 6**				**DAY 8**				**DAY 10**			
**Pairwise Comparisons of TREATMENT**				**Pairwise Comparisons of TREATMENT**				**Pairwise Comparisons of TREATMENT**			
Sample 1-Sample 2	Test Statistic	Sig.	Adj. Sig.[a]	Sample 1-Sample 2	Test Statistic	Sig.	Adj. Sig.[a]	Sample 1-Sample 2	Test Statistic	Sig.	Adj. Sig.[a]
PLA-PLATEC	6,000	0,014	0,043	PLA-PLATEC	6,000	0,014	0,043	PLA-PLATEC	0,667	0,414	1,000
PLA-PT10CVAC	6,000	0,014	0,043	PLA-PT10CVAC	6,000	0,014	0,043	PLA-PT10CVAC	6,000	0,014	0,043
PLATEC-PT10CVAC	6,000	0,014	0,043	PLATEC-PT10CVAC	6,000	0,014	0,043	PLATEC-PT10CVAC	0,667	0,414	1,000
Each row tests the null hypothesis that the Sample 1 and Sample 2 distributions are the same. Asymptotic significances (2-sided tests) are displayed. The significance level is ,050.				Each row tests the null hypothesis that the Sample 1 and Sample 2 distributions are the same. Asymptotic significances (2-sided tests) are displayed. The significance level is ,050.				Each row tests the null hypothesis that the Sample 1 and Sample 2 distributions are the same. Asymptotic significances (2-sided tests) are displayed. The significance level is ,050.			
a. Significance values have been adjusted by the Bonferroni correction for multiple tests.				a. Significance values have been adjusted by the Bonferroni correction for multiple tests.				a. Significance values have been adjusted by the Bonferroni correction for multiple tests.			

Table S7. Pearson's Correlation between TBARS and Heme-iron content during storage time.

Correlations							
		DAY0	DAY2	DAY4	DAY6	DAY8	DAY10
DAY0	Pearson Correlation	1	0,415	0,387	0,353	0,248	0,301
	Sig. (2-tailed)		0,087	0,113	0,151	0,322	0,224
	N	18	18	18	18	18	18
DAY2	Pearson Correlation	0,415	1	,999**	,995**	,971**	,979**
	Sig. (2-tailed)	0,087		0,000	0,000	0,000	0,000
	N	18	18	18	18	18	18
DAY4	Pearson Correlation	0,387	,999**	1	,997**	,980**	,984**
	Sig. (2-tailed)	0,113	0,000		0,000	0,000	0,000
	N	18	18	18	18	18	18
DAY6	Pearson Correlation	0,353	,995**	,997**	1	,988**	,984**
	Sig. (2-tailed)	0,151	0,000	0,000		0,000	0,000
	N	18	18	18	18	18	18
DAY8	Pearson Correlation	0,248	,971**	,980**	,988**	1	,979**
	Sig. (2-tailed)	0,322	0,000	0,000	0,000		0,000
	N	18	18	18	18	18	18
DAY10	Pearson Correlation	0,301	,979**	,984**	,984**	,979**	1
	Sig. (2-tailed)	0,224	0,000	0,000	0,000	0,000	
	N	18	18	18	18	18	18

**. Correlation is significant at the 0.01 level (2-tailed).

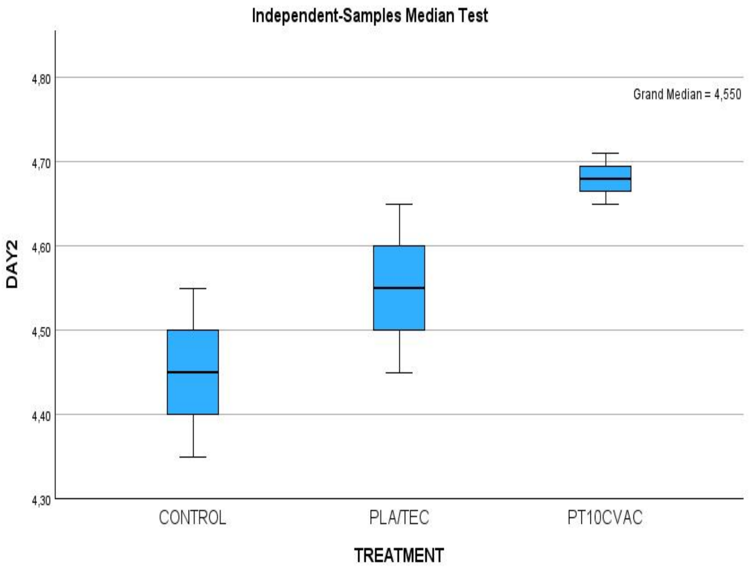
Independent-Samples Median Test
Grand Median = 4,550
DAY2
4,80
4,70
4,60
4,50
4,40
4,30
CONTROL
PLA/TEC
PT10CVAC
TREATMENT

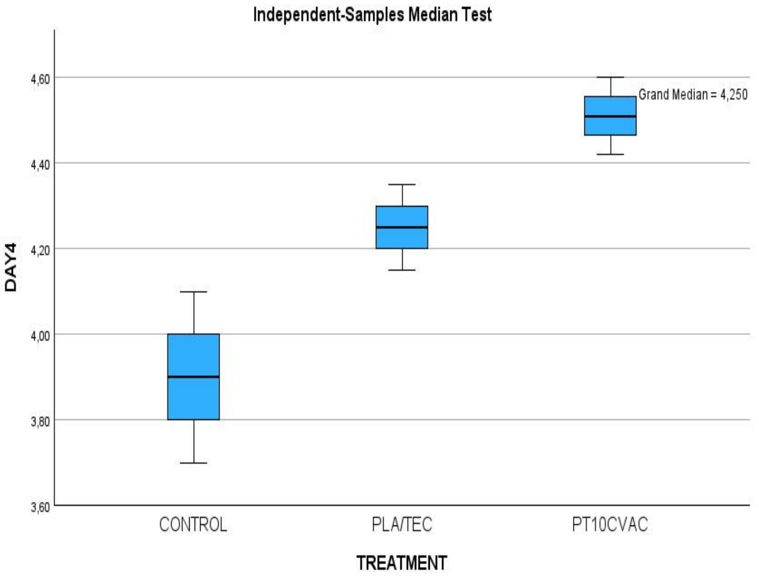
Independent-Samples Median Test
Grand Median = 4,250
DAY4
4,60
4,40
4,20
4,00
3,80
3,60
CONTROL
PLA/TEC
PT10CVAC
TREATMENT

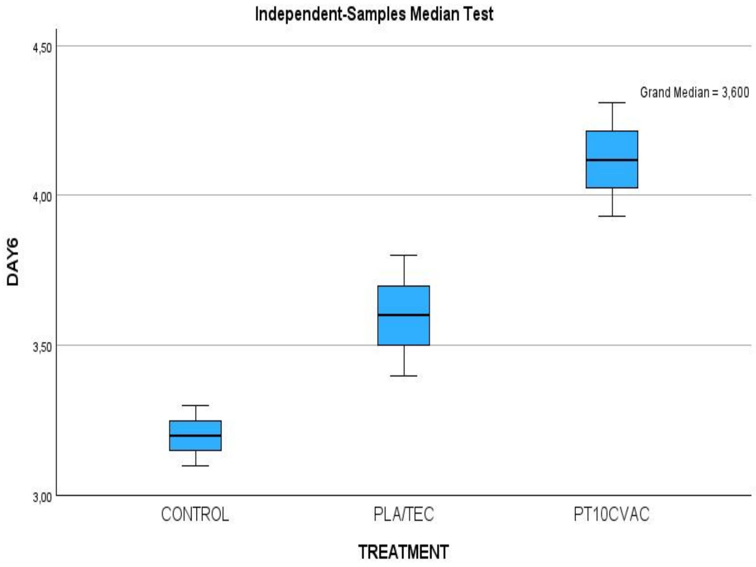
Independent-Samples Median Test
Grand Median = 3,600
DAY6
4,50
4,00
3,50
3,00
CONTROL
PLA/TEC
PT10CVAC
TREATMENT

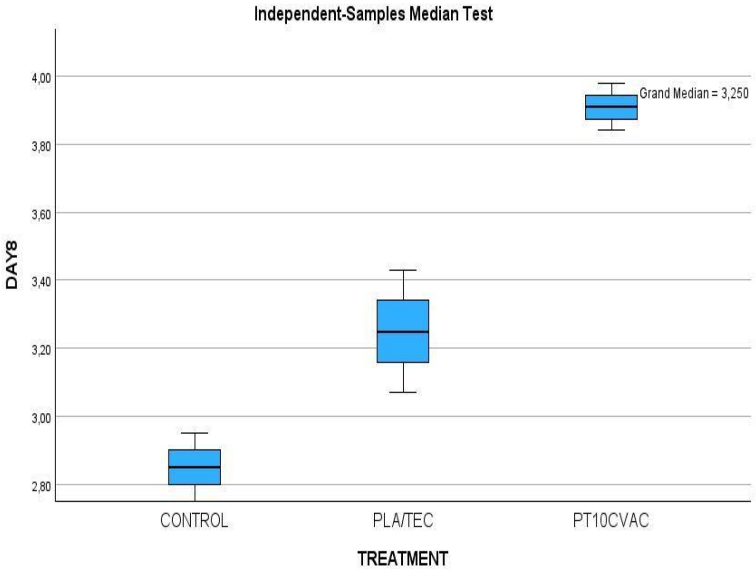
Independent-Samples Median Test
Grand Median = 3,250
DAY8
4,00
3,80
3,60
3,40
3,20
3,00
2,80
CONTROL
PLA/TEC
PT10CVAC
TREATMENT

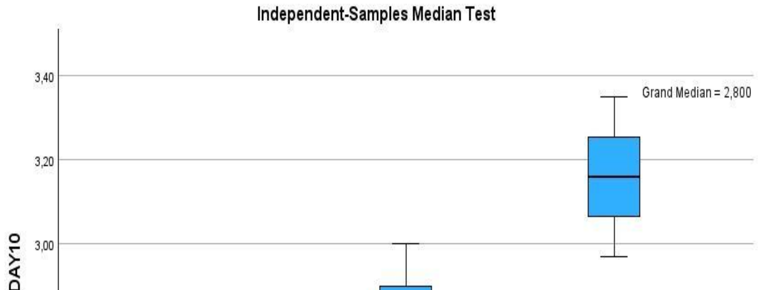
Independent-Samples Median Test
Grand Median = 2,800
DAY10
3,40
3,20
3,00

Figure S10. Independent-Samples Median Test of Odor durring storage time.

Table S8. Pairwise Comparisons of the different treatments according to the mean values of Odor during storage time.

**DAY 2**				**DAY 4**				**DAY 6**			
**Pairwise Comparisons of TREATMENT**				**Pairwise Comparisons of TREATMENT**				**Pairwise Comparisons of TREATMENT**			
Sample 1-Sample 2	Test Statistic	Sig.	Adj. Sig.[a]	Sample 1-Sample 2	Test Statistic	Sig.	Adj. Sig.[a]	Sample 1-Sample 2	Test Statistic	Sig.	Adj. Sig.[a]
CONTROL-PLA/TEC	0,667	0,414	1,000	CONTROL-PLA/TEC	6,000	0,014	0,043	CONTROL-PLA/TEC	6,000	0,014	0,043
CONTROL-PT10CVAC	6,000	0,014	0,043	CONTROL-PT10CVAC	6,000	0,014	0,043	CONTROL-PT10CVAC	6,000	0,014	0,043
PLA/TEC-PT10CVAC	3,000	0,083	0,250	PLA/TEC-PT10CVAC	6,000	0,014	0,043	PLA/TEC-PT10CVAC	6,000	0,014	0,043
Each row tests the null hypothesis that the Sample 1 and Sample 2 distributions are the same. Asymptotic significances (2-sided tests) are displayed. The significance level is ,050.				Each row tests the null hypothesis that the Sample 1 and Sample 2 distributions are the same. Asymptotic significances (2-sided tests) are displayed. The significance level is ,050.				Each row tests the null hypothesis that the Sample 1 and Sample 2 distributions are the same. Asymptotic significances (2-sided tests) are displayed. The significance level is ,050.			
a. Significance values have been adjusted by the Bonferroni correction for multiple tests.				a. Significance values have been adjusted by the Bonferroni correction for multiple tests.				a. Significance values have been adjusted by the Bonferroni correction for multiple tests.			

**DAY 8**				**DAY 10**			
**Pairwise Comparisons of TREATMENT**				**Pairwise Comparisons of TREATMENT**			
Sample 1-Sample 2	Test Statistic	Sig.	Adj. Sig.[a]	Sample 1-Sample 2	Test Statistic	Sig.	Adj. Sig.[a]
CONTROL-PLA/TEC	6,000	0,014	0,043	CONTROL-PLA/TEC	3,000	0,083	0,250

CONTROL-PT10CVAC	6,000	0,014	0,043	CONTROL-PT10CVAC	6,000	0,014	0,043
PLA/TEC-PT10CVAC	6,000	0,014	0,043	PLA/TEC-PT10CVAC	0,667	0,414	1,000
Each row tests the null hypothesis that the Sample 1 and Sample 2 distributions are the same. Asymptotic significances (2-sided tests) are displayed. The significance level is ,050.				Each row tests the null hypothesis that the Sample 1 and Sample 2 distributions are the same. Asymptotic significances (2-sided tests) are displayed. The significance level is ,050.			
a. Significance values have been adjusted by the Bonferroni correction for multiple tests.				a. Significance values have been adjusted by the Bonferroni correction for multiple tests.			

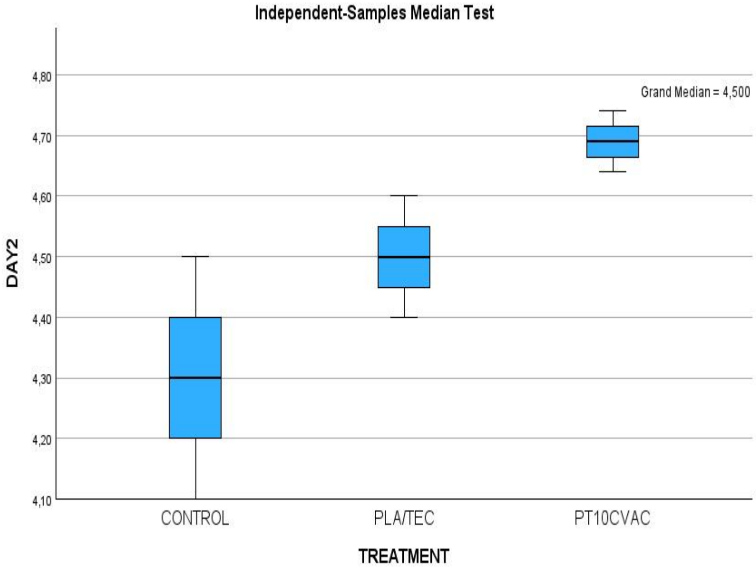

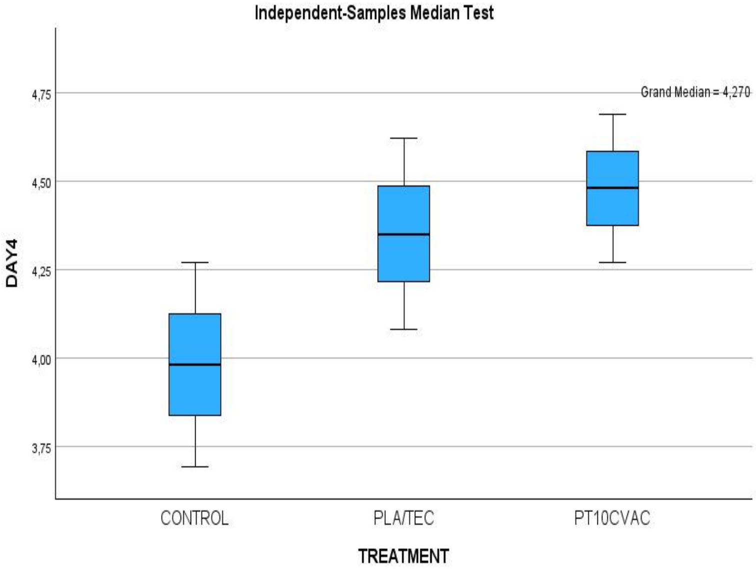

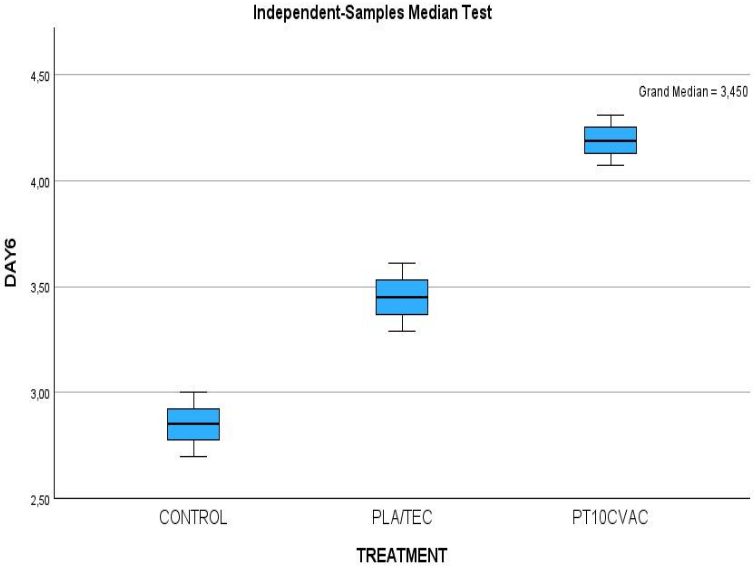

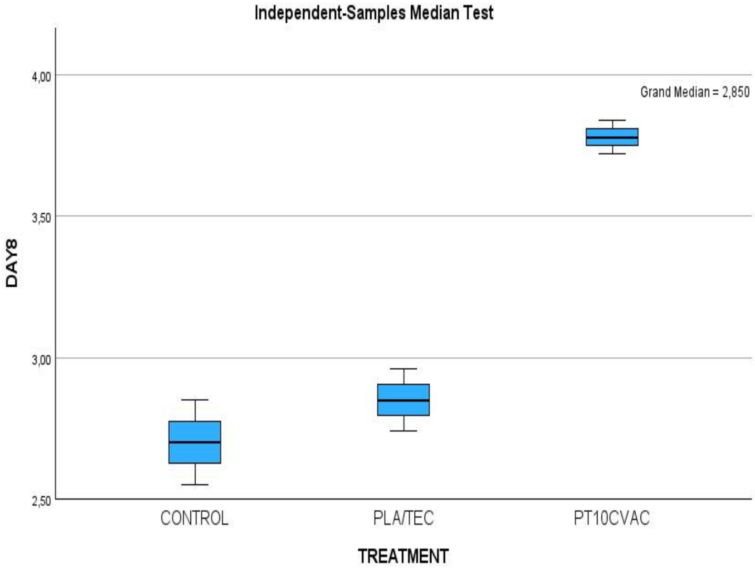

Figure S11. Independent-Samples Median Test of Color durring storage time.

Table S9. Pairwise Comparisons of the different treatments according to the mean values of Color during storage time.

DAY 2 — **Pairwise Comparisons of TREATMENT**

Sample 1-Sample 2	Test Statistic	Sig.	Adj. Sig.[a]
CONTROL-PLA/TEC	0,667	0,414	1,000
CONTROL-PT10CVAC	6,000	0,014	0,043
PLA/TEC-PT10CVAC	6,000	0,014	0,043

Each row tests the null hypothesis that the Sample 1 and Sample 2 distributions are the same. Asymptotic significances (2-sided tests) are displayed. The significance level is ,050.

a. Significance values have been adjusted by the Bonferroni correction for multiple tests.

DAY 4 — **Pairwise Comparisons of TREATMENT**

Sample 1-Sample 2	Test Statistic	Sig.	Adj. Sig.[a]
CONTROL-PLA/TEC	0,667	0,414	1,000
CONTROL-PT10CVAC	3,000	0,083	0,250
PLA/TEC-PT10CVAC	0,667	0,414	1,000

Each row tests the null hypothesis that the Sample 1 and Sample 2 distributions are the same. Asymptotic significances (2-sided tests) are displayed. The significance level is ,050.

a. Significance values have been adjusted by the Bonferroni correction for multiple tests.

DAY 6 — **Pairwise Comparisons of TREATMENT**

Sample 1-Sample 2	Test Statistic	Sig.	Adj. Sig.[a]
CONTROL-PLA/TEC	6,000	0,014	0,043
CONTROL-PT10CVAC	6,000	0,014	0,043
PLA/TEC-PT10CVAC	6,000	0,014	0,043

Each row tests the null hypothesis that the Sample 1 and Sample 2 distributions are the same. Asymptotic significances (2-sided tests) are displayed. The significance level is ,050.

a. Significance values have been adjusted by the Bonferroni correction for multiple tests.

DAY 8 — **Pairwise Comparisons of TREATMENT**

Sample 1-Sample 2	Test Statistic	Sig.	Adj. Sig.[a]
CONTROL-PLA/TEC	0,667	0,414	1,000
CONTROL-PT10CVAC	6,000	0,014	0,043
PLA/TEC-PT10CVAC	6,000	0,014	0,043

Each row tests the null hypothesis that the Sample 1 and Sample 2 distributions are the same. Asymptotic significances (2-sided tests) are displayed. The significance level is ,050.

a. Significance values have been adjusted by the Bonferroni correction for multiple tests.

DAY 10 — **Pairwise Comparisons of TREATMENT**

Sample 1-Sample 2	Test Statistic	Sig.	Adj. Sig.[a]
CONTROL-PLA/TEC	0,667	0,414	1,000
CONTROL-PT10CVAC	6,000	0,014	0,043
PLA/TEC-PT10CVAC	6,000	0,014	0,043

Each row tests the null hypothesis that the Sample 1 and Sample 2 distributions are the same. Asymptotic significances (2-sided tests) are displayed. The significance level is ,050.

a. Significance values have been adjusted by the Bonferroni correction for multiple tests.

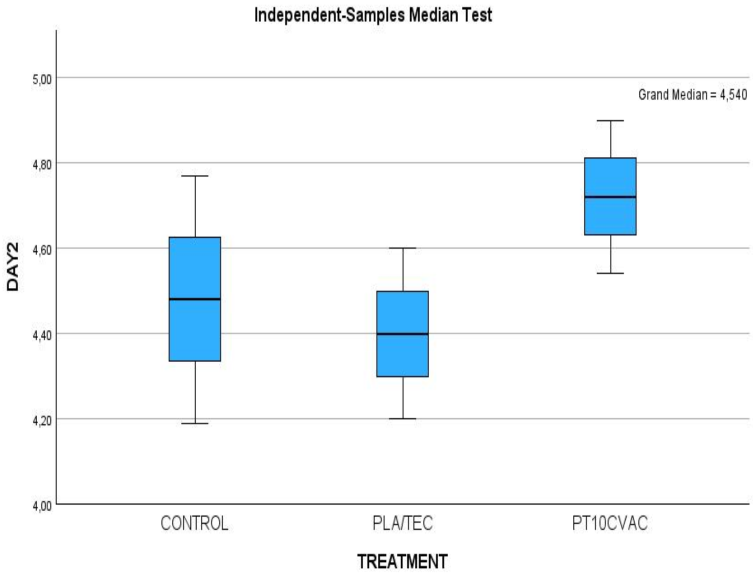
Independent-Samples Median Test
5,00
4,80
4,60
4,40
4,20
4,00
DAY2
Grand Median = 4,540
CONTROL
PLA/TEC
PT10CVAC
TREATMENT

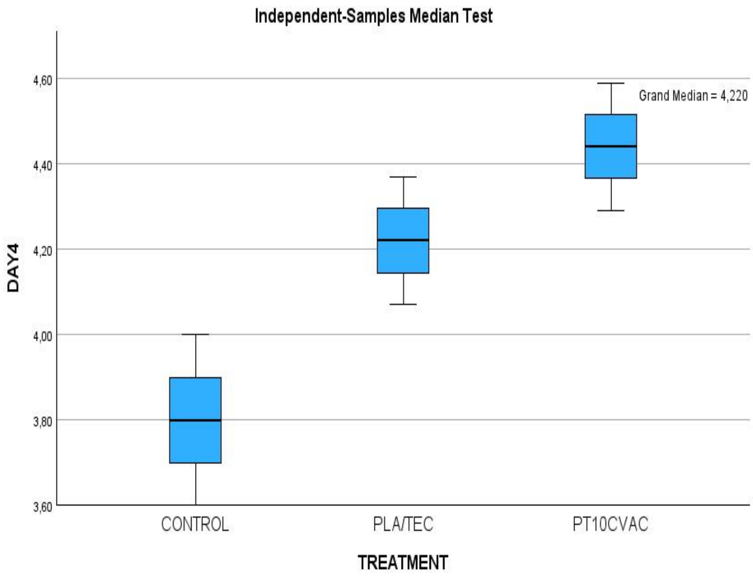
Independent-Samples Median Test
4,60
4,40
4,20
4,00
3,80
3,60
DAY4
Grand Median = 4,220
CONTROL
PLA/TEC
PT10CVAC
TREATMENT

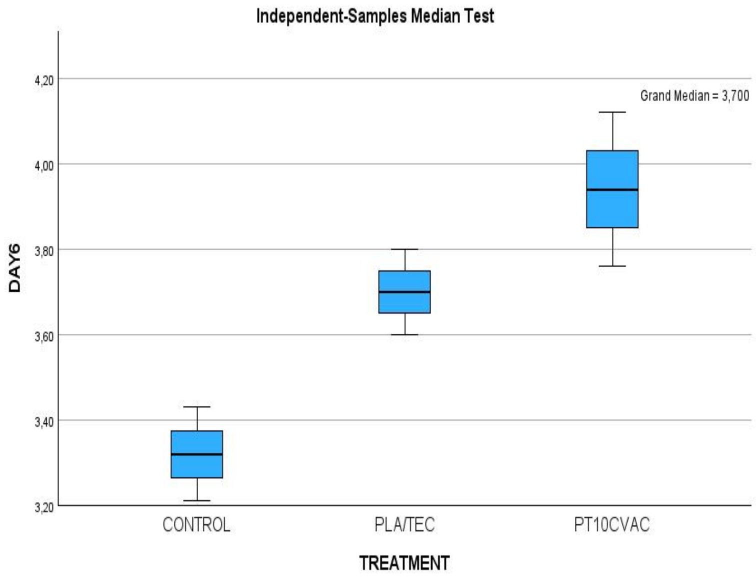
Independent-Samples Median Test
4,20
4,00
3,80
3,60
3,40
3,20
DAY6
Grand Median = 3,700
CONTROL
PLA/TEC
PT10CVAC
TREATMENT

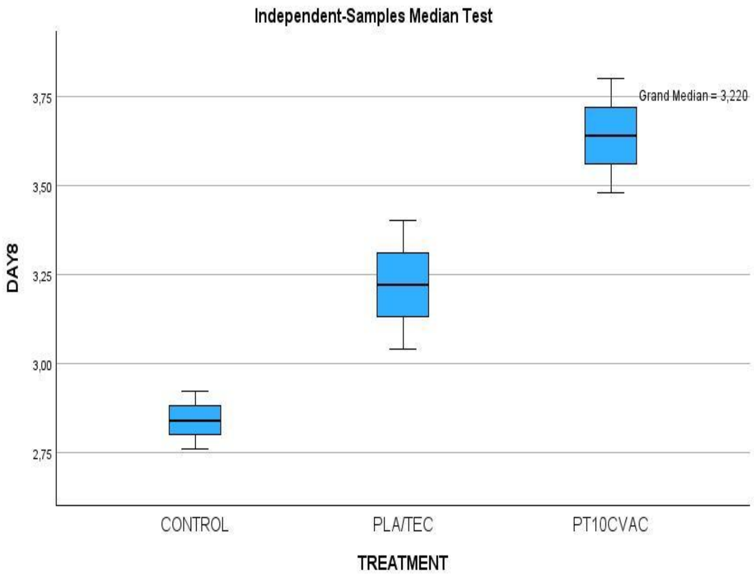
Independent-Samples Median Test
3,75
3,50
3,25
3,00
2,75
DAY8
Grand Median = 3,220
CONTROL
PLA/TEC
PT10CVAC
TREATMENT

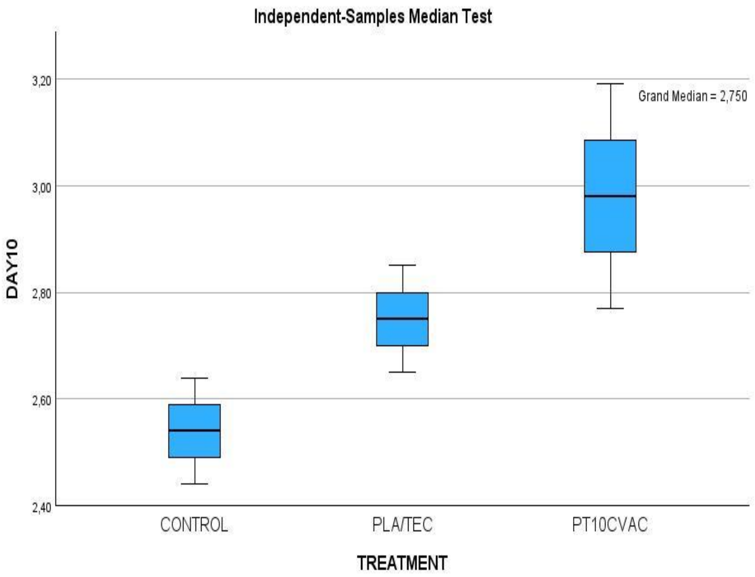

Figure S12. Independent-Samples Median Test of Texture durring storage time.

Table S10. Pairwise Comparisons of the different treatments according to the mean values of Texture
during storage time.

DAY 2				DAY 4				DAY 6			
**Pairwise Comparisons of TREATMENT**				**Pairwise Comparisons of TREATMENT**				**Pairwise Comparisons of TREATMENT**			
Sample 1-Sample 2	Test Statistic	Sig.	Adj. Sig.[a]	Sample 1-Sample 2	Test Statistic	Sig.	Adj. Sig.[a]	Sample 1-Sample 2	Test Statistic	Sig.	Adj. Sig.[a]
PLA/TEC-CONTROL	0,667	0,414	1,000	CONTROL-PLA/TEC	6,000	0,014	0,043	CONTROL-PLA/TEC	6,000	0,014	0,043
PLA/TEC-PT10CVAC	0,667	0,414	1,000	CONTROL-PT10CVAC	6,000	0,014	0,043	CONTROL-PT10CVAC	6,000	0,014	0,043
CONTROL-PT10CVAC	0,667	0,414	1,000	PLA/TEC-PT10CVAC	0,667	0,414	1,000	PLA/TEC-PT10CVAC	0,667	0,414	1,000
Each row tests the null hypothesis that the Sample 1 and Sample 2 distributions are the same. Asymptotic significances (2-sided tests) are displayed. The significance level is ,050.				Each row tests the null hypothesis that the Sample 1 and Sample 2 distributions are the same. Asymptotic significances (2-sided tests) are displayed. The significance level is ,050.				Each row tests the null hypothesis that the Sample 1 and Sample 2 distributions are the same. Asymptotic significances (2-sided tests) are displayed. The significance level is ,050.			
a. Significance values have been adjusted by the Bonferroni correction for multiple tests.				a. Significance values have been adjusted by the Bonferroni correction for multiple tests.				a. Significance values have been adjusted by the Bonferroni correction for multiple tests.			

DAY 8				DAY 10			
**Pairwise Comparisons of TREATMENT**				**Pairwise Comparisons of TREATMENT**			
Sample 1-Sample 2	Test Statistic	Sig.	Adj. Sig.[a]	Sample 1-Sample 2	Test Statistic	Sig.	Adj. Sig.[a]
CONTROL-PLA/TEC	6,000	0,014	0,043	CONTROL-PLA/TEC	6,000	0,014	0,043
CONTROL-PT10CVAC	6,000	0,014	0,043	CONTROL-PT10CVAC	6,000	0,014	0,043

PLA/TEC-PT10CVAC	6,000	0,014	0,043	PLA/TEC-PT10CVAC	0,667	0,414	1,000
Each row tests the null hypothesis that the Sample 1 and Sample 2 distributions are the same. Asymptotic significances (2-sided tests) are displayed. The significance level is ,050.				Each row tests the null hypothesis that the Sample 1 and Sample 2 distributions are the same. Asymptotic significances (2-sided tests) are displayed. The significance level is ,050.			
a. Significance values have been adjusted by the Bonferroni correction for multiple tests.				a. Significance values have been adjusted by the Bonferroni correction for multiple tests.			
